# High-Throughput Screening of a Marine Compound Library Identifies Anti-*Cryptosporidium* Activity of Leiodolide A

**DOI:** 10.3390/md20040240

**Published:** 2022-03-30

**Authors:** Rachel M. Bone Relat, Priscilla L. Winder, Gregory D. Bowden, Esther A. Guzmán, Tara A. Peterson, Shirley A. Pomponi, Jill C. Roberts, Amy E. Wright, Roberta M. O’Connor

**Affiliations:** 1 Department of Veterinary Microbiology and Pathology, College of Veterinary Medicine, Washington State University, 100 Dairy Rd, Pullman, WA 99164, USA; rachel.bone@wsu.edu (R.M.B.R.); gregory.bowden@wsu.edu (G.D.B.); 2 Harbor Branch Oceanographic Institute, Florida Atlantic University, 5600 US Highway 1 North, Fort Pierce, FL 34946, USA; pwinder@fau.edu (P.L.W.); eguzman9@fau.edu (E.A.G.); tpitts3@fau.edu (T.A.P.); spomponi@fau.edu (S.A.P.); jrober90@fau.edu (J.C.R.); 3 Department of Veterinary and Biomedical Sciences, College of Veterinary Medicine, University of Minnesota, 1971 Commonwealth Ave, St Paul, MN 55108, USA

**Keywords:** *Cryptosporidium*, high-throughput screen, anti-protozoal compounds, marine natural products, apicomplexan, leiodolide A

## Abstract

*Cryptosporidium* sp. are apicomplexan parasites that cause significant morbidity and possible mortality in humans and valuable livestock. There are no drugs on the market that are effective in the population most severely affected by this parasite. This study is the first high-throughput screen for potent anti-*Cryptosporidium* natural products sourced from a unique marine compound library. The Harbor Branch Oceanographic Institute at Florida Atlantic University has a collection of diverse marine organisms some of which have been subjected to medium pressure liquid chromatography to create an enriched fraction library. Numerous active compounds have been discovered from this library, but it has not been tested against *Cryptosporidium parvum*. A high-throughput in vitro growth inhibition assay was used to test 3764 fractions in the library, leading to the identification of 23 fractions that potently inhibited the growth of *Cryptosporidium parvum*. Bioassay guided fractionation of active fractions from a deep-sea sponge, *Leiodermatium* sp., resulted in the purification of leiodolide A, the major active compound in the organism. Leiodolide A displayed specific anti-*Cryptosporidium* activity at a half maximal effective concentration of 103.5 nM with selectivity indexes (SI) of 45.1, 11.9, 19.6 and 14.3 for human ileocecal colorectal adenocarcinoma cells (HCT-8), human hepatocellular carcinoma cells (Hep G2), human neuroblastoma cells (SH-SY5Y) and green monkey kidney cells (Vero), respectively. The unique structure of leiodolide A provides a valuable drug scaffold on which to develop new anti-*Cryptosporidium* compounds and supports the importance of screening natural product libraries for new chemical scaffolds.

## 1. Introduction

*Cryptosporidium* sp. is a zoonotic apicomplexan parasite that infects the gastrointestinal epithelium of its host. It is the leading cause of waterborne diarrheal disease in the United States [1,2] and the world [3] in both humans and valuable livestock [4]. *Cryptosporidium* is especially devastating in children as it causes life-threatening diarrhea with estimated annual deaths ranging in tens to hundreds of thousands [5,6]. Additionally, it is associated with developmental delays in children, including growth stunting and cognitive impairment [5]. While immunocompetent adults clear *Cryptosporidium* infection in days to weeks, immunocompromised individuals fail to resolve the infection, and systemic involvement and fatal diarrhea may develop in this population [7,8]. *Cryptosporidium* completes its entire life cycle within intestinal epithelial cells, first undergoing three rounds of asexual replication followed by development of gametes which fuse to produce oocysts that are then released into the environment [9,10]. This process leads to intestinal dysfunction and malabsorptive diarrhea [10]. No effective treatment exists for most human and all veterinary patients diagnosed with cryptosporidiosis [7,11]. Nitazoxanide is the only FDA drug approved to treat human cryptosporidiosis, but its use is limited as it is ineffectual in immunocompromised patients and not approved for use in children <1 year in age [7,12,13]. Currently, no drug exists that is effective in both immunocompetent and immunocompromised individuals [14]. 

The past decade has seen the identification of numerous lead anti-*cryptocry* compounds [14]. These new compounds have been discovered either via screens of known compounds or by targeting proteins known to be important for survival of related parasites. This approach reduces the likelihood of discovering new compound scaffolds. Recent data demonstrate that *Cryptosporidium*, like other apicomplexans, can rapidly develop drug resistance, even in a small experimental trial [15]. This observation underscores the need for a more robust drug discovery pipeline that will provide new scaffolds for anti-*Cryptosporidium* drugs. 

The marine environment is a rich source of compounds with potent biologic activity such as alkaloids, terpenes, polyketides, acetogenins, and peptides [16]. Marine natural products that inhibit the growth of *Plasmodium* sp., *Toxoplasma gondii*, *Leishmania* sp., and *Trypanosoma* sp. have been reported [17,18,19]. The potential to find anti-*Cryptosporidium* compounds from marine sources was recently highlighted by the discovery of tartrolon E, a broad spectrum anti-apicomplexan compound produced by a symbiotic bacterium of marine clams. Tartrolon E has nanomolar activity against *C. parvum* growth in vitro and is also highly effective in vivo [19]. To expand on this result, we screened a portion of the Harbor Branch Oceanographic Institute at Florida Atlantic University (HBOI) enriched fraction library for anti-*Cryptosporidium* compounds.

Screening of the HBOI fraction library has resulted in the discovery of numerous active natural products, including potent anti-*Plasmodium* compounds [17,20,21]. This library is derived from marine macroorganisms (algae and invertebrates) collected from diverse marine habitats, including deep and shallow reefs, sponge flats, mangroves, and artificial structures such as pier pilings and shipwrecks. A fractionation process using medium pressure liquid chromatography was used to create a highly enriched fraction library [22]. From screening a portion of the library for anti-*Cryptosporidium* activity, our group identified 23 fractions from eight taxonomically distinct organisms that potently inhibit the growth of *C. parvum*. This manuscript describes the identification and biological activity of the previously reported compound, leiodolide A [23,24] as the major anti-*Cryptosporidium* compound present in an enriched fraction derived from a deep-water *Leiodermatium* collected off the coast of the Bahamas. Leiodolide A was one of the most potent anti-*Cryptosporidium* compounds found from our phenotypic screen.

## 2. Results

**Figure 1:**
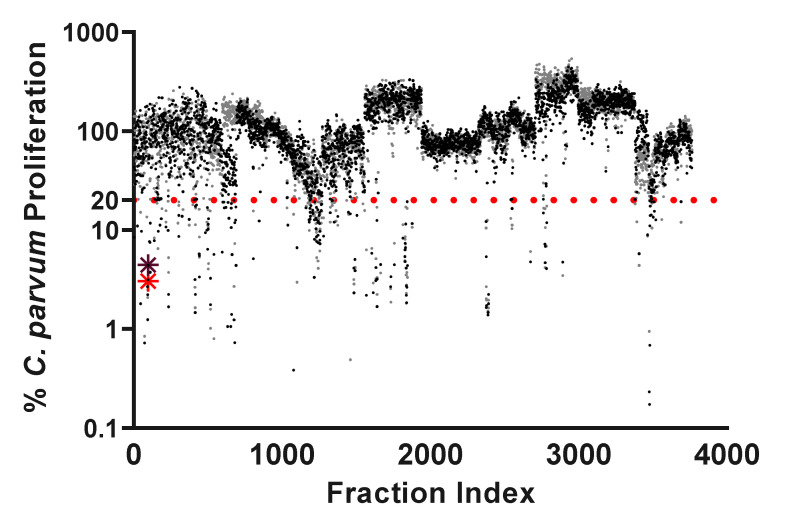
Scatter plot of the anti-*Cryptosporidium* activity of 3764 fractions from the HBOI library. All enriched fractions were screened at 10 ug/mL. A cut-off of 80% inhibition of *C. parvum* proliferation (dotted red line) in at least one of the two tested wells was applied to yield 222 hits for a hit rate of 5.8%. All points are normalized to a 0.1% DMSO vehicle control and log_10_ transformed. Red and purple asterisks mark the location of the fraction from which leiodolide A was isolated.

A high-throughput assay based on previously published methodology [25], was modified and utilized to identify fractions from the Harbor Branch Oceanographic Institute (HBOI) fraction library which have in vitro activity against *C. parvum.* The HBOI fraction library has been successfully screened against many targets and has yielded novel compounds that have activity against a range of cellular functions and diverse pathogens including the related apicomplexan *Plasmodium falciparum* [17,20,21]. In total, 3764 highly enriched fractions were screened for inhibition of *C. parvum* intracellular replication. Of the 3764 fractions screened, 222 decreased parasite proliferation by 80% or more in at least one of two technical replicates, when compared to a normalized DMSO vehicle control (Figure 1).

**Figure 2:**
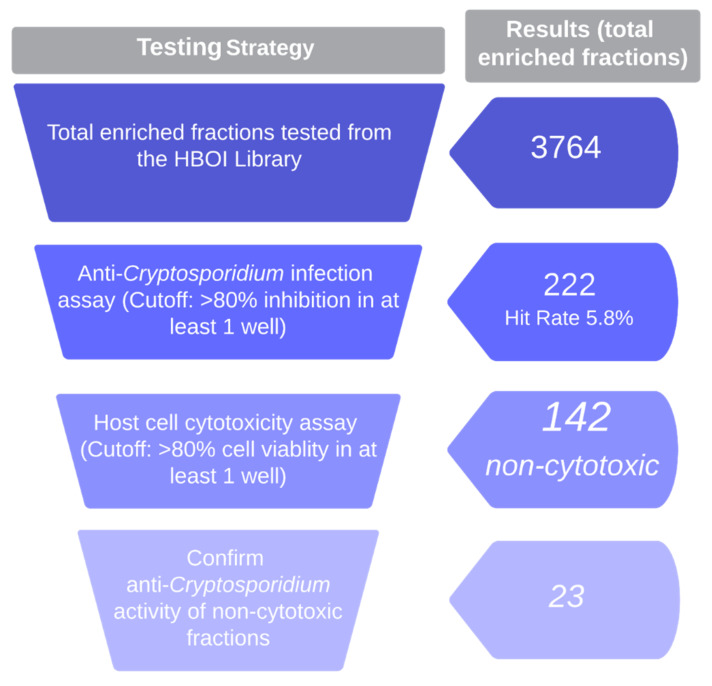
Testing strategy and results of high-throughput phenotypic screen of HBOI enriched fraction library for non-cytotoxic, anti-*Cryptosporidium* fractions. All fractions were tested at 10 μg/mL.

Four fractions were dropped from further screening due to visual observation of significant host cell cytotoxicity. Quantification of cellular viability by ATP measurement identified 76 fractions with cytotoxic activity against the HCT-8 host cells the parasites were grown in (Figure 2). The remaining non-cytotoxic fractions were retested to confirm inhibition of parasite growth and 23 anti-*Cryptosporidium* hits were identified (Figure 2).

These 23 fractions were derived from eight unique marine specimens including sponges and tunicates (List of fractions, Table S1). Work is on-going to identify the active compounds in these fractions. Five fractions from a *Leiodermatium* sp. showed strong activity. Fraction (HBOI.02.C07) was selected for further analysis due to the purity of the fraction, initial NMR data, and >95% *C. parvum* inhibition in both wells during the high-throughput phenotypic screen (described in Tables S1 and S2, Figure S1). 

**Figure 3:**
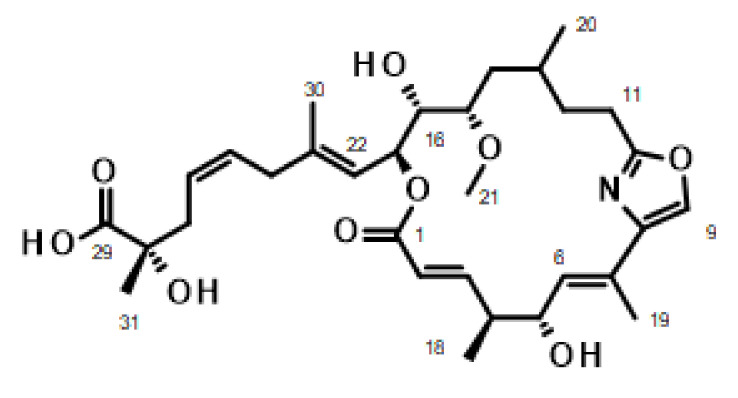
Published structure of leiodolide A [23].

This fraction was prepared through medium pressure flash chromatography of an ethyl acetate partition using an Isco Teledyne Combiflash^®^ Rf 4x instrument on a C-18 Rf gold column eluted with a gradient of acetonitrile in water (described in Table S3). The weight of available fraction was small, and therefore additional frozen sample was extracted and taken through the same chromatographic steps to yield a fraction that was ~95% pure. In our hands, leiodolide A had limited solubility in methanol, precipitating out of solution during overnight collection of the NMR data. Therefore, it was dried and transferred to *d_6_*-DMSO to collect a full 1D and 2D NMR data set (Figures S3–S14). Inspection of the ^13^C NMR spectrum coupled with the high resolution mass spectrometry (Figure S15) data suggested a molecular formula of C_31_H_45_NO_9_ for leiodolide A ([M + Na)]^+^ *m*/*z* observed 598.2986, calculated 598.2992, Δ = 0.6 mmu), indicating the presence of ten degrees of unsaturation. ^13^C NMR resonances attributable to a carboxylic acid (δ_C_ 177.7), two heterosubstituted sp^2^-hybridized carbons (δ_C_ 164.8 and δ_C_ 164.6), and additional olefinic carbons [δ_C_ 150.7(CH), 142.2(C), 140.6(C), 134.6(CH), 131.7(CH), 129.2(CH), 126.7(CH), 123.6(CH), 123.5(CH), 122.9(CH)] accounted for eight degrees of unsaturation suggesting that leiodolide A is bicyclic. The planar structure of leiodolide A was established from the analysis of its one- and two-dimensional NMR data. Interpretation of the 2D gCOSY spectrum coupled with the edited gHSQC spectrum led to the assignment of four isolated spin systems (C-2→C-6 with CH_3_-18; C-11→C15 with CH_3_-20; C-16→C-22 and C24→27). Analysis of ^2^J and ^3^J ^1^H-^13^C couplings observed in the gHMBC spectrum enabled these spin systems to be connected to the remaining atoms (Figure S2). A search of the literature revealed that the NMR and HRMS data are very similar and consistent with that reported previously for leiodolide A [23,24] (Figure 3). Assignment of the ^1^H NMR spectrum collected in *d_4_*-methanol and comparison to the published data confirmed the assignment of leiodolide A (Table S4, Figures S3–S15). 

**Figure 4:**
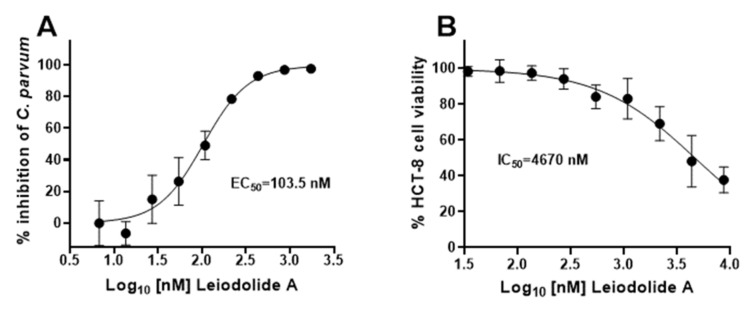
Leiodolide A inhibits *C. parvum* growth at nanomolar concentrations without affecting host cells. (**A**) EC_50_ of leiodolide A for intracellular *C. parvum* is 103.5 (95% CI 82.9–131.7) nM. HCT-8s were infected with *C. parvum* for 24 h and then infected cells were treated with the compound for 48 h. Data shown are the results of three independent experiments performed in triplicate. (**B**) IC_50_ of leiodolide A for confluent HCT-8 cells treated for 48 h is 4670 (95% CI 3640–6070) nM giving a selectivity index of 45.1. Data were normalized to a 0.1% DMSO control. The results of four independent experiments performed in triplicate are shown.

Testing of varying concentrations of leiodolide A in the *Cryptosporidium* infection assay yielded an EC_50_ of 103.5 (95% CI 82.9–131.7) nM in HCT-8 cells (Figure 4A). Cytotoxicity of leiodolide A against confluent HCT-8s was evaluated to confirm that the decrease in parasite growth seen was due to activity against the parasite and not due to host cell cytotoxicity. The concentration of leiodolide A at which host cell viability was 50% of a DMSO normalized control (IC_50_) for confluent HCT-8 cells treated for 48 h is 4670 (95% CI 3640–6070) nM producing a selectivity index (SI) of 45.1 (Figure 4B). The cytotoxicity of leiodolide A for Hep G2, Vero, and SH-SY5Y cell lines was also evaluated, and the IC_50_ and SI was calculated for each (Table 1). 

## 3. Discussion

To our knowledge, this is the first study to screen a marine-derived library to search for novel and potent anti-*Cryptosporidium* compounds. Leiodolide A is only the second marine-derived compound identified thus far that inhibits *C. parvum* growth in vitro, with the first being tartrolon E, derived from a symbiotic bacterium of marine mollusks [19]. Research to identify and develop marine natural products that can be used to treat *C. parvum* infection is nascent. However, efforts to source marine natural products effective against *Plasmodium* sp., a medically important related apicomplexan, and the causative agent of malaria, has been actively ongoing for decades. Marine natural products with anti-plasmodial activities have been discovered from a range of marine sources [17,20,26,27]. This previous success prompted our group to search for marine natural products with anti-*Cryptosporidium* activity. In this study, we observed that leiodolide A, a marine natural product previously isolated from a deep-water *Leiodermatium* collected off the coast of Palau [23,24], exhibited potent inhibition of *C. parvum* growth. When tested against the National Cancer Institute’s 60 cell line panel, leiodolide A had significant cytotoxic activity against HL-60 leukemia and OVCAR-3 ovarian cancer cell lines (average GI_50_ 2.0 μM) [23]. In our study, the compound exhibited good selectivity for the parasite over the host cells with an SI of 45.1, indicating that leiodolide A is a promising new anti-*Cryptosporidium* candidate. 

The successful transition from a drug in discovery and pre-clinical stages to FDA approval is a heroic task. It is estimated that over 90% of new compounds that are deemed safe and efficacious during pre-clinical research ultimately fail during clinical trial [28]. Given this failure rate, more than 10 compounds would need to enter clinical trials for one drug to be approved. While there are several promising anti-*Cryptosporidium* compounds in the pipeline, there are not enough to guarantee success in clinical trials. Currently there are three compounds that have been extensively tested in animal models. A piperazine-based drug (MMV665917) was discovered via screening the Medicines for Malaria Venture malaria box and shown to be effective against infection in immunocompromised mice, and against cryptosporidiosis in calves and pigs [29,30,31]. The PI(4)K(phosphatidylinositol-4-OH kinase) inhibitor (pyrazolopyridine KDU731) was identified in a large scale, high-throughput phenotypic screen of the Novartis Institute for Tropical Diseases (NITD) parasite box and shown to prevent diarrhea in infected neonatal calves [32]. The bumped kinase inhibitors of the *Cryptosporidium parvum* calcium-dependent protein kinase 1 (cpCDK1), identified by targeted screens of protein kinase inhibitors [33,34] have shown success in multiple animal models of disease [35,36]. However, pre-clinical success does not guarantee clinical success. In screening a drug repurposing library, clofazimine, an FDA approved drug for leprosy treatment, was discovered to have significant anti-*Cryptosporidium* activity, and this drug performed well in pre-clinical studies [37]. However, in a Phase 2A clinical trial, clofazimine failed to meet primary end goals to demonstrate efficacy, and the trial was terminated [38]. Thus, while there are promising drugs in the pipeline, screens focused on identifying diverse sources of compounds and scaffolds are important to enhance the robustness of this pipeline. For example, the discovery of leiodolide A’s anti-*Cryptosporidium* activity provides a novel scaffold for development of even more effective derivatives.

Leiodolide A is a 19-membered macrolide, probably made through a mixed polyketide (PKS) and non-ribosomal peptide synthase (NRPS) biosynthesis. It contains an embedded oxazole as found in other marine-derived macrolides such as the kabiramides, derived from related sponges. Marine derived macrolides have shown a variety of biological activities ranging from cytotoxic, antibacterial, antifungal, antimitotic, antiviral, and antiplasmodial properties [39]. Unexpectedly, the isolation of leiodolide A reported here yielded material with different solubility characteristics than that reported by Sandler et al. [23,24]. It showed limited solubility in *d*_4-_methanol. In *d*_6_-DMSO, several resonances in the NMR spectra were very broad, with the most notable being those for the C-25–C-26 double bond and the quaternary carbon C-28. Upon further purification to remove minor impurities prior to further biological testing, the ^1^H NMR spectrum for leiodolide A showed very broad to non-existent resonances for the C-22 to C-29 side chain, with only small resonances for the C-30 methyl protons and very broad resonances for H-25, H-26 and H-27. Additionally, H-17 was also reduced in size and broadened (Figure S5). Leiodolide A shares a terminal α-hydroxy-α-methyl carboxylic acid moiety with okadaic acid for which the NMR spectra have been reported to be broadened after purification by reverse phase HPLC. This broadening has been ascribed to binding of K^+^ ion and has been demonstrated to be necessary for biological activity as uncomplexed okadaic acid shows greatly reduced activity in a rat uterine contraction assay [40]. Okadaic acid has also been shown to form oligomers around the K^+^ ion which are thought to assist with transport across cell membranes [41,42,43]. It is possible that leiodolide A also binds metal ions such as K^+^ leading to the broadened/missing resonances observed in this study.

While the SI in host cells is 45.1, the SI of leiodolide A in Hep G2 cells is 11.9. This SI is narrow, and an increase in SI is likely needed to move forward with this compound. An increase in the SI may be achievable via synthesized analogs and derivatives. Synthesis of improved analogs was undertaken in the development and refinement of the triazolopyridazine, MMV665917, an anti-*Cryptosporidium* compound which showed promising in vivo and in vitro efficacy but had the potential for moderate cardiac cytotoxicity [44]. In this instance, the synthesis and testing of analogs led to the identification of a compound that was more potent with an EC_50_ of 0.17 μM versus 2.1 μM for the original compound, with an improved cytotoxicity profile. Similarly, bumped kinase inhibitors of cpCDK1 were discovered that have potent in vitro and in vivo parasite growth inhibition, but were considered liabilities for cardiotoxicity due to off target effects [33,34]. Through the synthesis and identification of analogs, the in vitro and in vivo safety profile of the lead bumped kinase inhibitors has significantly improved [36]. These ongoing drug discovery efforts highlight the importance of drug scaffolds as a starting place for future discovery and safety assessment ventures needed for the successful development of an effective and safe anti-*Cryptosporidium* drug.

There has been substantial interest in the synthesis of leiodolide A, and several groups have made significant progress towards the synthesis of portions of the molecule while one nominal synthesis has been completed [45,46,47,48,49,50]. Questions on the overall stereochemical configuration of the molecule remain. In the nominal synthesis of leiodolide A reported by Edenharter, the NMR spectra are not identical to the natural product with a significant difference observed for the ^1^H chemical shift of CH_3_-18, resulting in a suggested revision to 4R, 5R configuration from the tentative assignment of 4S, 5R made by Sandler et al. In the current isolation, the 5-OH proton was observed in the spectra collected in *d6*-DMSO and showed correlations in the 2D-NOESY experiment to H-3, H-5, H-6, and H_3_-18. Inverting the configuration of H-5 to S places the CH_3_-18 in a pseudoequatorial position and the 5-hydroxy group on the top face of the molecule, and is consistent with all observed nOe correlations (Table S4, Figures S2 and S10–S14). Data that does not match is the large ^3^J_C,H_ reported by Sandler et al., which requires H-5 and CH_3_-18 to be anti-periplanar to each other [51]. The macrolide ring has significant flexibility and final assignment awaits completion of a total synthesis. The identification of potent anti-*Cryptosporidium* activity for leiodolide A may encourage the synthetic organic community to prepare the additional diastereomers and production of analogs with improved selectivity for the parasite. The syntheses completed to date are well poised to achieve this. The Fürstner group completed the total synthesis for various diastereomers of the related compound leiodolide B, but none of them matched the NMR data reported for the natural product [52]. Leiodolide B was not detected in the active fractions identified in this study.

## 4. Materials and Methods

### 4.1. Extraction/Formation of Enriched Fractions 

The creation of the Harbor Branch Oceanographic Institute (HBOI) at Florida Atlantic University enriched fraction library has been described previously [22]. Briefly, the library was generated by extraction of chemically rich frozen specimens from the HBOI marine specimen collection [53], followed by fractionation by either medium pressure liquid chromatography using an Isco Combiflash^®^ purification system or vacuum column chromatography (Teledyne Isco, Lincoln, NE, USA,). The choice of stationary phases for each separation was based upon high performance liquid chromatography (HPLC) analysis of the extracts and most were fractionated using C-18 reverse phase chromatography or normal phase chromatography on silica gel. Enriched fractions are stored dry at −20 °C until plated for assay. 

### 4.2. Handling of Fraction Library

An amount of 100 μg of each enriched fraction was plated into a separate well of a 96-well plate, and the solvent was removed for shipping to the O’Connor laboratory. Prior to assay each well was resuspended in 100% dimethyl sulfoxide (DMSO) to create a 10 mg/mL solution. Plates were maintained at −80 °C, and only removed for assay dilution preparation. 

### 4.3. C. parvum Strains and Storage

Transgenic nanoluciferase-expressing *Cryptosporidium parvum* Iowa II strain oocysts [54] were obtained from the Sterling Laboratory at the University of Arizona. Parasites were stored at 4 °C and used within four months of receipt.

### 4.4. HCT-8 Cell Culture

Human ileocecal colorectal adenocarcinoma cells (HCT-8 cells; ATCC CCL244) were maintained and subcultured according to the ATCC’s recommended procedures to a maximum of 30 passages.

### 4.5. High-Throughput Phenotypic Screen

A high-throughput screen to find inhibitors of *C. parvum* growth in HCT-8 host cells was performed according to a previously published methodology [25] with a few modifications. Briefly, HCT-8 intestinal cells (ATCC CCL244) were subcultured in complete medium according to manufacturer’s recommendations, and seeded into 96-well, clear bottom, white-walled cell culture plates (Greiner Bio-One, Monroe, NC, USA). Cells were grown until 90 to 100% confluent then infected with 10,000 nano-luciferase oocysts per well pretreated with bleach as previously described [25]. To optimize excystation and infection, oocysts were added to cells in complete medium supplemented with 0.6% taurocholic acid. Assay plates were centrifuged at 1000× *g* for 5 min, and infection allowed to proceed for 24 h. To prepare fractions for testing, dried fractions were dissolved in 100% DMSO to give stock solutions of 10 mg/mL. Fractions were further diluted in complete media to give a final concentration of 10 μg/mL (0.1% DMSO) and added to the infected cells. At 72 h post infection (48 h post treatment), plates were centrifuged, media was aspirated and cells were lysed in fecal lysis buffer [25], for one hour. Nano-Glo luciferase reagent (Promega, Madison, WI, USA) was then added to each well and the total luminescence of each well was read on a BiokTek Synergy HTX plate reader (Agilent, CA, USA). Percent inhibition of parasite growth was calculated as [(RLU_DMSO_ − RLU_treatment_)/RLU_DMSO_] × 100 where RLU is relative luciferase units. Controls for this experiment were a vehicle control of cells treated with 0.1% DMSO, a positive control of cells treated with 100 ng/mL of tartrolon E [19], and a negative control of untreated cells Each fraction was tested in duplicate on separate plates run concurrently. Fractions that inhibited parasite growth by >80% on either one (“single hit”) or both (“double hit”) plates were selected for further testing. In a few instances, visual assessment of the monolayer, prompted by a color change in the culture medium, identified fractions that were highly toxic to host cells, and these were excluded from further analysis. 

### 4.6. Initial HCT-8 Cytotoxicity Assay

Each fraction that inhibited parasite growth was evaluated for cytotoxicity against HCT-8 host cells by quantifying cellular ATP (Cell-Titer Glo 2.0, Promega, Madison, WI, USA). This assay was performed according to previously published methodology [25]. Briefly, HCT-8s were seeded onto a 96-well plate and allowed to reach 25–50% confluence. After this level of confluence was achieved (24–36 h), fractions considered hits in the previous screen were diluted in infection media and added to the plate at 10 μg/mL. Plates were incubated for 48 h, after which Cell-Titer Glo 2.0 reagent was added, and plates were read on a BiokTek Synergy HTX plate reader. A vehicle control of 0.1% DMSO and negative control of HCT-8 cells in complete media were included on each plate. Cell viability was calculated as % cell viability = [(RLU_treatment_)/RLU_DMSO_] × 100. Fractions were run in triplicate and those that exhibited >80% viability were moved forward for additional testing.

### 4.7. Confirmatory Testing in C. parvum Infection Assay

Non-cytotoxic fractions that inhibited *C. parvum* growth were retested, as described in the infection assay, to confirm anti-parasitic activity.

### 4.8. Collection of the Marine Specimen Used to Purify Leiodolide A

A specimen of sponge identified as *Leiodermatium* sp. (Phylum: Porifera, Class: Demospongiae, Subclass Heteroscleromorpha, Order Tetractinellida, Suborder Spirophorina, Family Azoricidae); HBOI Marine Biotechnology Research Collection Sample ID: 14-X-03-1-004, was collected on 14 October 2003 using the Johnson Sea Link I manned submersible at a depth of 571 m on a shell hash slope off Hogsty Reef, Bahamas (Latitude 21°40′18.4″ N Latitude 73°50′50.6″ W) and stored at −20 °C until workup (Figure 3). A full description of the sponge can be found in Table S2 and Figure S1. 

### 4.9. Isolation and Structure Elucidation of Leiodolide A

The preparation of the original highly enriched fraction that showed activity in the primary screen can be found in Table S3. The purification of material used in the secondary biological and chemical studies is as follows. The frozen sponge (393 g) was pulverized with a hammer, and then exhaustively extracted with ethanol followed by ethyl acetate:ethanol (9:1 *v*/*v*) using a Waring blender. The combined filtered extract was distilled under reduced pressure to yield 8.55 g of crude extract. The residue was partitioned between H_2_O (100 mL), and ethyl acetate (300 mL × 3) and after concentration by distillation under reduced pressure, yielded 750 mg of organic partition. The organic partition was further purified using reverse-phase flash chromatography on a Teledyne Isco CombiFlash^®^ Rx4 equipped with PeakTrak software (Teledyne Isco, Lincoln, NE, USA) as follows: 740 mg of partition was absorbed onto C-18 packing and then loaded into a loading column. Separation was conducted on a 15.5 g Teledyne Isco Rf Gold C18 column operating at a flow rate of 30 mL/min, monitored at 225 and 270 nm, and collected into 13 mm tubes. Solvent A was H_2_O:CH_3_CN (95:5), Solvent B was CH_3_CN, Solvent C was CH_3_OH, and Solvent D was CH_2_Cl_2_. The run lasted 28.4 min over 55.6 column volumes. The column was first eluted with a mixture of A:B (9:1) for 4 column volumes. The column was then eluted over a linear gradient to 100% B over 31.6 column volumes and then held at 100% B for 6.7 column volumes. The column was then washed with 100% solvent C (MeOH) for 3.3 column volumes followed by a linear gradient to 100% solvent D (CH_2_Cl_2_) over 10 column volumes. The column was then washed with H_2_O:CH_3_CN:TFA (1:4:0.1%). A fraction eluting between column volumes 20–22 (designated as fraction 6, 14.3 mg) showed activity in the screening assay and was approximately 95% pure. This material was used in the initial NMR analysis to identify the structure as leiodolide A and for much of the testing. Fraction 6 was further purified by HPLC on a Phenomenex Onyx monolithic C18 column 10 mm × 100 mm (Phenomenex, Torrance, CA, USA) with a linear gradient as follows: Solvent A: H_2_O:CH_3_CN (95:5 *v*/*v*), Solvent B: CH_3_CN; *t* = 0 min, A:B 90:10; *t* = 12 min, A:B 40:60; *t* = 14 min, 100% B; *t* = 18 min, 100% B. Highly pure leiodolide A (1.1 mg) eluted as a broad peak between 6 and 9 min.

NMR data were collected on a JEOL ECA-600 spectrometer (JEOL USA, Peabody, MA, USA) operating at 600.17 MHz for ^1^H and 150.9 MHz for ^13^C. The edited-g-HSQC spectrum was optimized for 140 Hz, the g-HMBC spectrum was optimized for 8 Hz, and the band selective g-HMBC experiment was optimized for 8 Hz. Chemical shifts were referenced to solvent, e.g., *d_6_*-DMSO δ_H_ observed at 2.50 ppm and δ_C_ observed at 39.51 ppm. The HRMS spectrum was measured using a JEOL AccuTOF-DART 4G (JEOL USA, Peabody, MA, USA) using a paper spray attachment. Interpretation of a full 1D and 2D NMR data set (1D: ^1^H, ^13^C, 2D: edited-gHSQC, g-COSY, g-HMBC and NOESY) coupled with the high resolution mass spectrometry data (HRMS) identified the compound as leiodolide A, a compound reported previously from a Palau collection of *Leiodermatium* sp. [23,24]. Additional purification of the material was conducted using reverse phase HPLC to remove minor impurities yielding >95% pure compound leiodolide A (Table S4, Figures S3–S15).

### 4.10. Determination of Half Maximal Effective Concentration (EC_50_)

Two-fold serial dilutions of purified leiodolide A were tested from 1738.1 to 6.8 nM against infected *C. parvum* HCT-8 cells as described in the infection assay. The percent growth inhibition of parasites treated at each concentration was normalized to a 0.1% DMSO vehicle control using the equation % inhibition = [(RLU_DMSO_-RLU_Treatment_)/RLU_DMSO_] × 100. Samples were run in triplicate and the averages from three independent experiments were recorded. These averages were used to calculate the half-maximal effective concentration (EC_50_) and 95% confidence interval using the log(inhibitor) versus response-variable slope (four-parameter) regression in GraphPad Prism 9.3 (GraphPad, La Jolla, CA, USA).

### 4.11. Host Cell Cytotoxicity under Experimental Conditions

As *C. parvum* is an obligate intracellular parasite, it was necessary to test the host cell cytotoxicity of leiodolide A under infection assay conditions to ensure that the compound was inhibiting parasite growth and not affecting host cells. Therefore, host cell toxicity of leiodolide A was determined via the Cell-Titer Glo 2.0 assay for ATP as previously described above, with the following changes: 96-well plates seeded with HCT-8 cells were allowed to reach confluence and then treated with a two-fold serial dilution of leiodolide A from 8690.8 to 33.9 nM. Samples were run in triplicate and the averages from four independent experiments were used to determine the concentration at which treated cells had 50% viability compared to a 0.1% DMSO control (IC_50_). The IC_50_ and 95% confidence interval were calculated using the log(inhibitor) versus response-variable slope (four-parameter) regression equation in GraphPad Prism 9.3 (GraphPad, La Jolla, CA, USA).

### 4.12. Cytotoxicity against Hep G2 Cells, Vero and SH-SY5Y Cells

#### 4.12.1. Cell Culture

The human hepatocellular carcinoma Hep G2 (ATCC^®^ HB-8065), the human neuroblastoma SH-SY5Y (ATCC^®^ CRL-2266), and the “normal” African Green Monkey Vero (ATCC^®^ CCL-81) cell lines were obtained from the American Type Culture Collection (ATCC, Manassas, VA, USA) and cultured following ATCC’s recommendations. Cells were maintained at 37 °C and 5% CO_2_ for a maximum of 20 passages when a new aliquot was thawed.

#### 4.12.2. Cytotoxicity Assay (MTT)

In total, 3000 cells/well were plated on a 384-well tissue culture plate at a volume of 30 μL/well and allowed to adhere overnight, when 30 μL of medium containing treatment were added. Treatment consisted of serial dilutions of the compound from 20 to 0.0098 μg/mL, media alone, or media with methanol (solvent control). The cells were incubated for 72 h at 37 °C and 5% CO_2_. After this incubation, 25 μL of a 5 mg/mL solution of 3-(4,5-Dimethyl-2-thiazolyl)-2,5-diphenyl-2H-tetrazolium bromide (MTT, Millipore Sigma (St. Louis, MO, USA)) were added to each well. Cells were incubated for 3 h at 37 °C. After centrifugation and removal of the supernatant, 100 μL of acidified isopropyl alcohol (1:500 hydrochloric acid to isopropanol solution) were added to each well. The crystals were dissolved by shaking or pipetting. The absorbency of each well was measured at 570 nm with a plate reader (NOVOstar, BMG Labtech Inc., Durham, NC, USA). The resulting absorbencies were normalized against the solvent control. The dose required to see 50% cell death (IC_50_) was calculated by changing the data to logarithm scale and subjecting it to a nonlinear regression using GraphPad 5.0 software (San Diego, CA, USA). Values shown represent the average from three independent experiments and the 95% confidence interval. 

## 5. Conclusions

*Cryptosporidium* is a devastating disease that lacks an effective treatment for many human patients and valuable livestock. Development of an effective therapeutic is a veterinary and medical imperative. Leiodolide A is extremely promising anti-*Cryptosporidium* compound with nM level activity against the parasite and a unique chemical structure. The discovery of this novel biological activity supports the utility of additional screening of marine natural products for anti-*Cryptosporidium* activity. Additionally, future studies to create synthetic analogs that improve the potency and selectivity of leiodolide A for the parasite is an important next step. 

## Figures and Tables

**Table 1:** The EC_50_, and IC_50_ of various cell lines treated with leiodolide A.

EC_50_ (nM) ^a^	IC_50_ (nM) ^a^							
*C. parvum* Cultured in HCT-8s	HCT-8	SI	Hep G2	SI	Vero	SI	SH-SY5Y	SI
103.5 (82.9–131.7)	4670 (3640–6070)	45.1	1228 (1136–1328)	11.9	1476 (1361–1601)	14.3	2025 (1776–2309)	19.6

^a^ Values are 95% confidence interval (in parentheses) from a minimum of three experiments.

## Data Availability

All data are contained within the manuscript or additional provided supplemental data. Original NMR data files are available upon request to AEW.

## Supplementary Materials

The following supporting information can be downloaded at: https://www.mdpi.com/article/10.3390/md20040240/s1. Table S1. List of 23 non-cytotoxic, confirmed enriched fractions discovered via the high-throughput screen. Table S2. Biological material used in the isolation of Leiodolide A. Figure S1. Pictures of *Leiodermatium* sp. used in the study and map of collection site. Table S3. Preparation of the enriched fractions identified as active in the primary screen for *Leiodermatium* sp. Figure S2. Structure of Leiodolide A and data supporting a possible revision of 5R configuration to 5S. Table S4. Comparison of published NMR data for Leiodolide A [23] and data from this isolation. Figure S3. ^1^H NMR spectrum of Leiodolide A used in the study (*d*_4_-methanol, 600 MHz). Figure S4. ^1^H NMR spectrum of Leiodolide A used in the study (*d*_6_-DMSO, 600 MHz). Figure S5. Comparison of ^1^H NMR spectra of Leiodolide A before and after further purification by reverse phase HPLC (*d*_4_-methanol, 600 MHz). Figure S6. ^13^C NMR spectrum of Leiodolide A used in the study (*d*_6_-DMSO, 150 MHz). Figure S7. 2D-edited g-HSQC spectrum of Leiodolide A used in the study (*d*_6_-DMSO, 600 MHz). Figure S8. 2D-HMBC spectrum of Leiodolide A used in the study (*d*_6_-DMSO, 600 MHz). Figure S9. 2D-COSY spectrum of Leiodolide A used in the study (*d*_6_-DMSO, 600 MHz). Figure S10. 2D-NOESY spectrum of Leiodolide A used in the study (*d*_6_-DMSO, 600 MHz). Figure S11. Expansion of 2D-NOESY spectrum of Leiodolide A used in the study (*d*_6_-DMSO, 600 MHz). Figure S12. Expansion of 2D-NOESY spectrum of Leiodolide A used in the study (*d*_6_-DMSO, 600 MHz). Figure S13. Expansion of 2D-NOESY spectrum of Leiodolide A used in the study (*d*_6_-DMSO, 600 MHz). Figure S14. Expansion of 2D-NOESY spectrum of Leiodolide A used in the study (*d*_6_-DMSO, 600 MHz). Figure S15. High resolution mass spectrum of Leiodolide A used in this study.
