# Supplementary material for: High-Throughput Screening of a Marine Compound Library Identifies Anti-Cryptosporidium Activity of Leiodolide A
Source: Mar Drugs. 2022 Mar 30;20(4):240. doi: 10.3390/md20040240 (PMC9026894; doi:10.3390/md20040240)
Supplement: Supplementary file 1 [file marinedrugs-20-00240-s001.zip › marinedrugs-1639084-supplementary.pdf]

Supplementary Information

Article

High-throughput Screening of a Marine Compound Library Identifies Anti-*Cryptosporidium* Activity of Leiodolide A

Rachel M. Bone Relat [1], Priscilla L. Winder [2], Gregory D. Bowden [1], Esther A. Guzmán [2], Tara A. Peterson [2], Shirley A. Pomponi [2], Jill C. Roberts [2], Amy E. Wright [2,*] and Roberta M. O'Connor [1,3,*]

[1] Department of Veterinary Microbiology and Pathology, College of Veterinary Medicine, Washington State University, 100 Dairy Rd, Pullman, Washington, WA 99164, USA; rachel.bone@wsu.edu (R.M.B.R.); Gregory.bowden@wsu.edu (G.D.B.)

[2] Harbor Branch Oceanographic Institute, Florida Atlantic University, 5600 US Highway 1 North, Fort Pierce, FL 34946, USA; pwinder@fau.edu (P.L.W.); eguzman9@fau.edu (E.A.G.); tpitts3@fau.edu (T.A.P.); spomponi@fau.edu (S.A.P.); jrober90@fau.edu (J.C.R.)

[3] Department of Veterinary and Biomedical Sciences, College of Veterinary Medicine, University of Minnesota, 1971 Commonwealth Ave, St Paul, MN 55108, USA

* Correspondence: awrigh33@fau.edu (A.E.W.); ocon0586@umn.edu (R.M.O.)

Table S1. List of 23 non-cytotoxic, confirmed enriched fractions discovered via the high-throughput screen.

Table S2. Biological material used in the isolation of Leiodolide A.

Figure S1. Pictures of *Leiodermatium* sp. used in the study and map of where it was collected.

Table S3. Preparation of the enriched fractions identified as active in the primary screen for *Leiodermatium* sp.

Figure S2. Structure of Leiodolide A, 1 and data supporting a possible revision of 5R configuration to 5S.

Table S4. Comparison of published NMR data for Leiodolide A (Sandler et. al JOC 2006, 71, 7245) and data from this isolation.

Figure S3. ^{1}H NMR spectrum of Leiodolide A used in the study (d_4-methanol, 600 MHz).

Figure S4. ^{1}H NMR spectrum of Leiodolide A used in the study (d_6-DMSO, 600 MHz).

Figure S5. Comparison of ^{1}H NMR spectra of Leiodolide A before and after further purification by reverse phase HPLC (*d4*-methanol, 600 MHz).

Figure S6. ^{13}C NMR spectrum of Leiodolide A used in the study (d_6-DMSO, 150 MHz).

Figure S7. 2D-edited g-HSQC spectrum of Leiodolide A used in the study (d_6-DMSO, 600 MHz).

Figure S8. 2D-HMBC spectrum of Leiodolide A used in the study (d_6-DMSO, 600 MHz).

Figure S9. 2D-COSY spectrum of Leiodolide A used in the study (d_6-DMSO, 600 MHz).
Figure S10. 2D-NOESY spectrum of Leiodolide A used in the study (d_6-DMSO, 600 MHz).
Figure S11. Expansion of 2D-NOESY spectrum of Leiodolide A used in the study (d_6-DMSO, 600 MHz).
Figure S12. Expansion of 2D-NOESY spectrum of Leiodolide A used in the study (d_6-DMSO, 600 MHz).
Figure S13. Expansion of 2D-NOESY spectrum of Leiodolide A used in the study (d_6-DMSO, 600 MHz).
Figure S14. Expansion of 2D-NOESY spectrum of Leiodolide A used in the study (d_6-DMSO, 600 MHz).
Figure S15. High resolution mass spectrum of Leiodolide A used in this study.

Table S1. List of 23 non-cytotoxic, confirmed enriched fractions discovered via the high-throughput screen. Fractions are derived from 8 unique species. The fraction in red was selected for further testing based on availability, purity of sample, % inhibition on the initial screen, % cell viability, and % inhibition during confirmation.

Sample ID	Higher Level Taxonomy of Organism	High-throughput Screen-% Inhibition nLuc *C. parvum* growth Replicate 1	High-throughput Screen-% Inhibition nLuc *C. parvum* growth Replicate 2	Host Cytotoxicity Assay- Average % HCT-8 viability	Inhibition Confirmation Assay-% Average *C. parvum* growth inhibition
HBOI.002.C04	Leiodermatium sp.	97.32	97.11	77.28	98.13
HBOI.002.C06	Leiodermatium sp.	98.76	97.51	92.05	97.58
HBOI.002.C07	Leiodermatium sp.	96.86	97.22	79.16	98.41
HBOI.002.C08	Leiodermatium sp.	95.56	96.95	88.77	88.82
HBOI.002.C09	Leiodermatium sp.	95.61	97.54	81.96	98.39
HBOI.004.B01	Plakinidae #1	90.10	80.45	80.18	86.40
HBOI.004.B04	Plakinidae #1	97.76	94.74	82.24	98.43
HBOI.005.F05	Plakinidae #2	87.21	88.81	96.86	79.32
HBOI.006.D02	Haplosclerida #1	96.65	94.70	91.53	92.51
HBOI.006.D03	Haplosclerida #1	94.49	91.52	91.80	85.29
HBOI.020.F06	Clavelinidae	91.73	88.69	84.26	98.15
HBOI.020.G08	Didemnidae #1	94.61	95.12	85.26	97.62
HBOI.020.G09	Didemnidae #1	93.72	95.18	84.95	97.88
HBOI.020.G10	Didemnidae #1	95.22	91.96	90.17	97.75
HBOI.020.H02	Didemnidae #1	88.52	82.91	89.06	97.91
HBOI.020.H05	Didemnidae #1	95.88	96.48	81.88	98.07
HBOI.020.H08	Didemnidae #1	98.15	97.22	80.20	97.96
HBOI.020.H09	Didemnidae #1	97.36	96.27	109.22	97.01
HBOI.021.A02	Didemnidae #1	95.64	96.67	103.13	97.73
HBOI.029.F03	Polycitoridae #1	95.23	93.92	109.57	94.66
HBOI.030.E07	Theonellidae #1	85.13	77.79	106.44	93.20
HBOI.030.E08	Theonellidae #1	84.90	83.32	102.15	94.66
HBOI.030.G04	Theonellidae #1	88.14	82.55	84.64	97.56

Table S2. Biological material used in the isolation of Leiodolide A.

**Leiodolide A used in this study**	**References for Taxonomic ID of *Leiodermatium* specimen:**
Leiodolide A used in this study was isolated from a specimen of the sponge *Leiodermatium* sp. The specimen was collected on October 14, 2003 using the Johnson Sea Link I manned submersible at a depth of 571 m on a shell hash slope off Hogsty Reef, Bahamas (Latitude 21°40′18.4″N Latitude 73°50′50.6″W) and stored at −20°C until workup (Marine Biotechnology Research Collection sample number: 14-X-03-1-004). The sponge is tan, brittle to hard in consistency, and its morphology is a thin-walled fluted plate. Its external morphology and spicules are characteristic of the genus *Leiodermatium* [1,2]; Phylum Porifera, Class Demospongiae, Subclass Heteroscleromorpha, Order Tetractinellida, Suborder Spirophorina, Family Azoricidae [3].	[1] Schmidt, O. *Grundzüge einer Spongien-Fauna des atlantischen Gebietes*; Wilhelm Engelmann: Leipzig, Germany, 1870; p. 22.<br><br>[2] Pisera, A.; Lévi, C. Family Azoricidae Sollas, 1888. In *Systema Porifera. A Guide to the Classification of Sponges*; Hooper, J.N.A., Van Soest, R.W.M., Eds.; Kluwer Academic/Plenum Publishers: New York, NY, USA, 2002; pp. 352–355.<br><br>[3] de Voogd, N.J.; Alvarez, B.; Boury-Esnault, N.; Carballo, J.L.; Cárdenas, P.; Díaz, M.-C.; Dohrmann, M.; Downey, R.; Hajdu, E.; Hooper, J.N.A.; Kelly, M.; Klautau, M.; Manconi, R.; Morrow, C.C. Pisera, A.B.; Ríos, P.; Rützler, K.; Schönberg, C.; Vacelet, J.; van Soest, R.W.M. World Porifera Database. *Leiodermatium lynceus* Schmidt, 1870. Available online: http://www.marinespecies.org/porifera/porifera.php?p=taxdetails&id=134344 on 2022-02-22 (accessed on 22 February 2022).

Figure S1. Pictures of *Leiodermatium* sp. used in the study and map of where it was collected. Source material for leiodolide A used in this study. (**A**) In situ photo of *Leiodermatium* sp. used in this study to identify leiodolide A as an anti-*Cryptosporidium* compound. (**B**) Map showing collection site for *Leiodermatium* sp. The red pointer indicates the collection site for the sponge off Hogsty Reef in the Bahamas From Google Maps, by Google: Google.com/maps/place 21°40′18.4″N + 73°50′50.6″W (Accessed 20 February 2022). (**C**) Deck photo of the sponge used in this study with ruler to provide scale of sample.

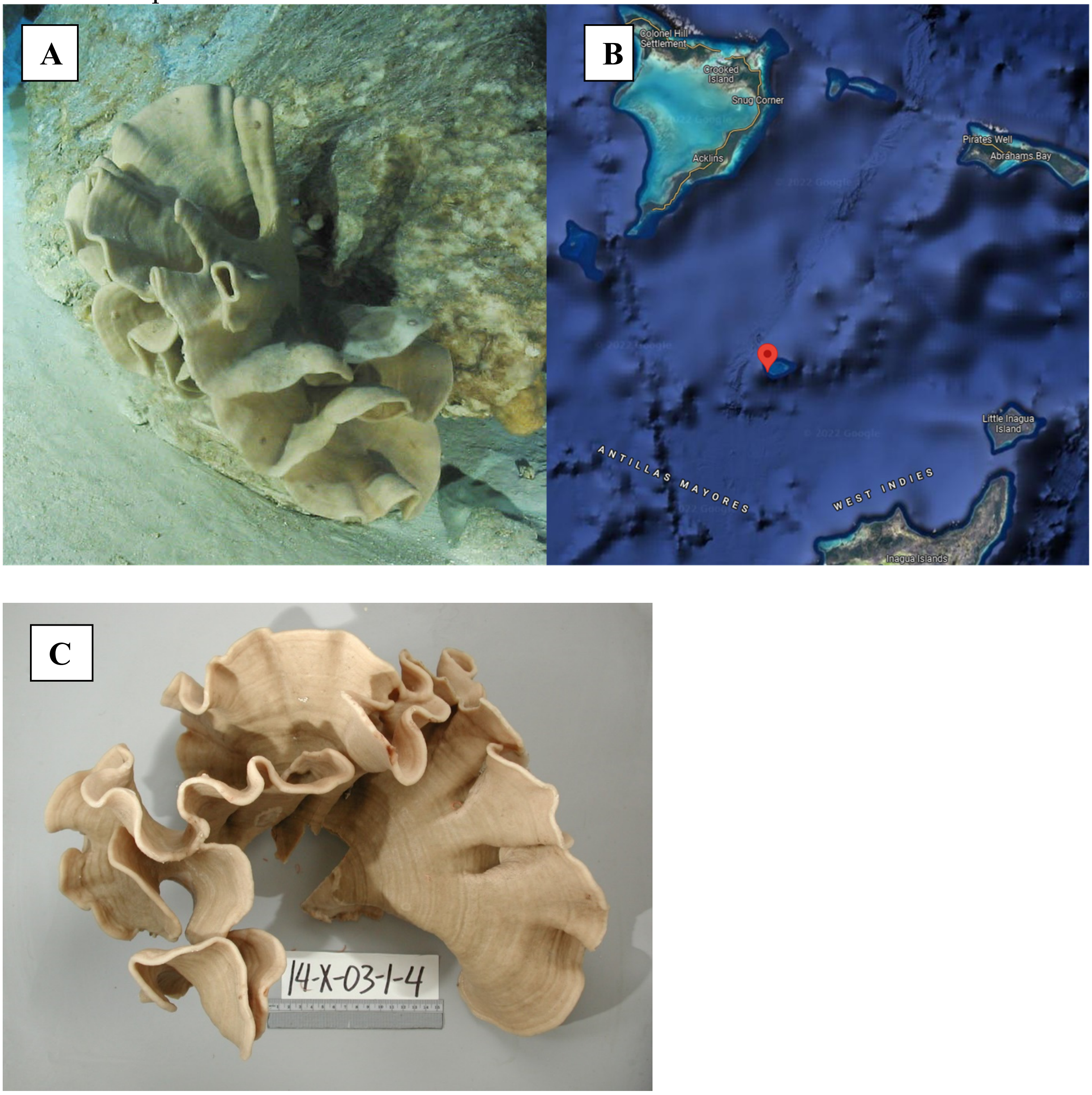

Table S3. Preparation of the enriched fractions identified as active in the primary screen for *Leiodermatium* sp.

Purification procedure for initial active fractions	Analytical data for leiodolide A used in this study
The initial library fractions from *Leiodermatium* sp. identified as active in the primary screen were prepared as follows: The frozen sponge (14-X-03-1-004), 300 g) was extracted by pulverizing with a hammer, then ground using a Waring blender with ethanol (200 mL X4), followed by ethyl acetate:ethanol (9:1 v/v; 250 mL X1). The combined filtered extract was concentrated by distillation under reduced pressure to yield 8.55 g of crude extract. The residue was partitioned between ethyl acetate (100 mL × 3) and water (100 mL), which after concentration by distillation under reduced pressure, yielded 683 mg of the ethyl acetate partition. The ethyl acetate partition was further purified using reverse-phase flash chromatography on a Teledyne Isco CombiFlash Rx4 equipped with PeakTrak software (Teledyne Isco, Lincoln, NE, USA) as follows: 647 mg of partition was loaded onto a 30g Teledyne Isco Rf Gold C18 column operating at a flow rate of 30 mL/min, monitored by UV absorbance at 225 and 270 nm and collected into 13 mm tubes. Solvent A was H_2O:CH_3CN (95:5), Solvent B was CH_3CN, Solvent C was CH_3OH, and Solvent D was CH_2Cl_2. The run lasted 15.9 mins and 37 column volumes (CV). The column was first eluted with a mixture of A:B (94:6) for 2.2 CV. The column was then eluted over a linear gradient to 100% B over 17.8 CV and then held at 100% B for 6 CV. The column was then washed with 100% solvent C (MeOH) for 1 CV followed by a linear gradient to 100% solvent D (CH_2Cl_2) over 5 CV then held at 100% D for 1 CV and then at 100% MeOH for 4 CV. A series of five active fractions eluting at 12-15 CV (tubes 23-28, HBOI.002.C04, 4.9 mg), 17-18 CV (tubes 32-34, HBOI.002.C06, 4.3 mg), 21-22 CV (tubes 38-40, HBOI.002.C07, 4.5 mg), 24-32 CV (tubes 41-56 HBOI.002.C08, 5.9 mg) and 32-35 CV (tubes 57-64, HBOI.002.C09,17.3 mg) were collected. Fraction HBOI.002.C07 contained leiodolide A.	Leiodolide A: clear oil solid; $[\alpha]^{24}_{D}$ +28.8 (*c* 0.52, MeOH); UV (MeOH) λ_{max} 229 nm; ^{1}H and ^{13}C NMR data, see Table S5; HRMS *m/z* $[M + Na]^+$ *m/z* 598.2986 (calcd for $C_{31}H_{45}NO_9Na$, 598.2992, Δ = 0.6mmu) The optical rotation was measured on a Rudolph Research Analytical Autopol III. The UV was measured on a Thermo Fisher Scientific Nanodrop.

Figure S2. Structure of Leiodolide A and data supporting a possible revision of 5R configuration to 5S. Bonds in red show the spin systems derived from 2D ^{1}H-^{1}H COSY. Arrows show the correlations in the 2D ^{1}H-^{13}C HMBC spectrum that tie the systems together. Arrows go from the coupled protons and end at the carbon.

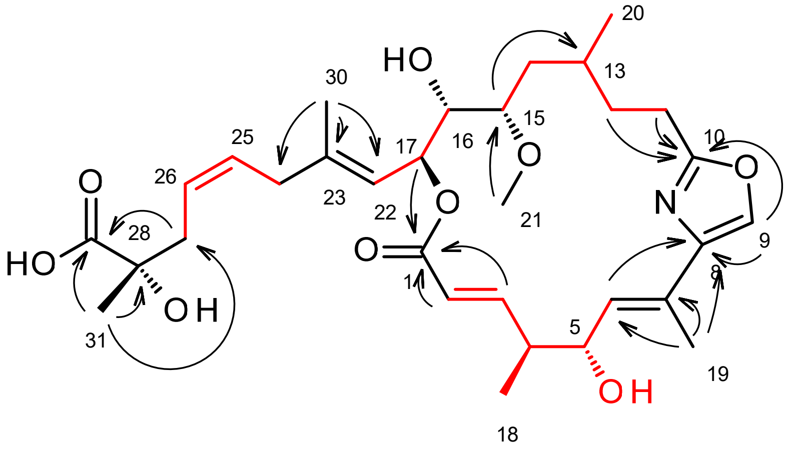

(1)

There has been significant progress towards the synthesis of leiodolide A with several groups synthesizing portions of the molecule and one nominal synthesis being completed. Questions on the overall stereochemical configuration of the molecule remain. In the nominal synthesis of leiodolide A reported by Edenharter [1], the NMR spectra are not identical to the natural product with a very significant difference observed for the ^{1}H chemical shift of CH_3-18 resulting in a suggested revision to 4R, 5R configuration from the tentative assignment of 4S, 5R made by Sandler et.al. In the current isolation, the 5-OH proton was observed in the spectra collected in *d6*-DMSO and showed correlations in the 2D-NOESY experiment to H-3, H-5, H-6 and H_3-18. Inverting the configuration of H-5 to S places the CH_3-18 in a pseudoequatorial position and the 5-hydroxy group on the top face of the molecule and allows for all observed nOe correlations to be explained (Table S4 Figures S10–S15). In assigning the S* stereochemistry for C-5, the Cahn-Ingold-Prelog priority of groups was assigned as follows: Priority 1: OH, Priority 2 C6 and priority 3: C-4 [both C6 and C-4 have a H, a C and a C by the Cahn-Ingold Rules, but C-7 has three C (is quaternary) while C-3 has an H, a C and C), thus C-6 was chosen as the higher priority]. Data that does not match this assignment of configuration is the large $^{3}J_{C,H}$ reported by Sandler et. al. between H-5 and C-18 which requires H-5 and CH_3-18 to be anti-periplanar according to the rules defined for acyclic systems by Matsumori et. al. [2]. The nOe data observed in this isolation is not consistent with them being placed anti to each other. It is possible that this isolation of leiodolide A has differing configuration, or that the phenomena that broadens some

resonances impacts the conformation, but most of the observed chemical shifts and coupling constants are similar. Unfortunately, the macrolide ring has significant flexibility and final assignment awaits completion of a total synthesis of the possible diastereomers. Sawhorse and Newman projections for this portion of the molecule are shown below.

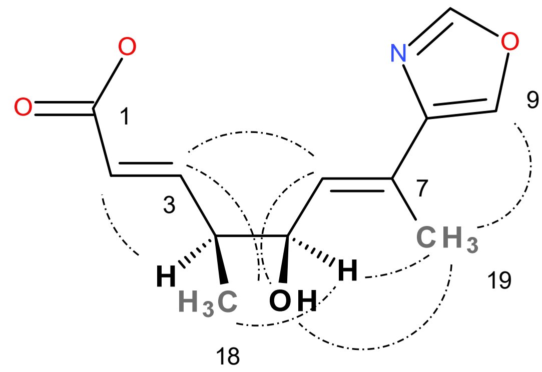

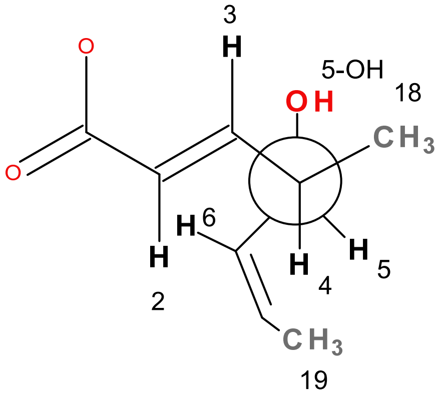

References:
[1] Edenharter, A. Total Synthesis of Nominal Leiodolide A and Semi synthesis of Deazaepothilone C. Doctoral thesis, ETH Zürich, Zürich, Switzerland, 2017. Available online: https://doi.org/10.3929/ethz-b-000216172 (accessed on 14 February 2022).
[2] Matsumori, N.; Kaneno, D.; Murata, M.; Nakamura, H.; Tachibana, K. Stereochemical Determination of Acyclic Structures Based on Carbon−Proton Spin-Coupling Constants. A Method of Configuration Analysis for Natural Products *J. Org. Chem.* **1999**, *64*, 866–876.

Table S4. Comparison of published NMR data for Leiodolide A (Sandler et. al JOC 2006, 71, 7245) and data from this isolation.

Published Leiodolide A d4-methanol				This isolation d4-methanol	This isolation d6-DMSO ^{1}H 600Mhz ; ^{13}C 150 MHz						
Atom		^{13}C	^{1}H	^{1}H	^{13}C		^{1}H		COSY	HMBC[b]	NOESY
1		166.9			164.8	C_q					
2		124.3	5.71 (d 15.5)	5.67 (d 15.5)	122.9	CH	5.62	d (15.5)	3	1, 4	3, 4, 5-OHw, 6
3		151.2	6.89 (dd 15.5, 9.5)	6.87 dd (15.8, 9.8)	150.7	CH	6.76	dd (15.8, 9.6)	2, 4	1,18	2, 4, 5-OH, 6, 18
4		44.9	2.39 m	2.35 m	43.0	CH	2.28	m (ddq (9.6, 2.75, 6.8)[a]	3, 5, 18	2, 3	2, 3, 5, 6(w), 18
5		72.3	4.55 (dd 8.5, 2.0)	4.52 (dd 8.9, 2.1)	69.9	CH	4.39	bddd (8.25, 2.75, 3.44)	4, 6		4, 5-OH, 6, 18, 19
5-OH							4.92	d (4.1)			3, 5, 6, 18
6		131.4	6.32 (dd 8.5, 1.5)	6.29 d (8.25)	131.7	CH	6.2	d (8.9)	5, 19	7, 8, 19	2, 3, 4, 5, 5-OH, 15, 21
7		125.6			123.5	C_q					
8		143.5			142.2	C_q					
9		135	7.68 s	7.65 (s)	134.6	CH	7.9	s	19?	8, 10	4(w), 19
10		166.3			164.6	C_q					
11	a	25.2	2.83 m	2.82 m	23.8	CH_2	2.81	dt (15.8, 6.87)	11b, 12ab	10, 12, 13	12a, 12b
	b		2.71 m	2.70 m			2.64	m	11a, 12ab	10, 12, 13	12a, 12b
12	a	34.7	2.05 m	2.01 m (6 lines)	32.9	CH_2	1.95	sextet?	11ab, 12b, 13	10, 11, 13, 20	11a, 11b, 12b, 13, 20
	b		1.60 m	1.56 m (6 lines)			1.46	sextet?	11ab, 12a, 13	10, 11, 13, 14, 20	11a, 11b, 12a, 15,20
13		29.5	1.39 m	1.26 ?(obscured)	28.1	CH	1.23	overlap	12ab, 14, 20		14, 15?, 16?
14	ab	35.9	1.62 m	1.62 t (6.4)	34.1	CH_2	1.52	m	13, 15	15	11a, 15, 16, 21
15		80.2	3.24 (ddd 8, 4.0, 0.5)	3.21 (t 6.9)	78.8	CH	3.1	m	14	13, 21	6w?, 12a, 12b, 13?, 14, 17, 20
16		73.5	3.46 (d 9.5)	3.42 (d 8.9)	71.6	CH	3.38	under H2O	17	17, 22	
17		70.8	5.77 (t 9.5)	5.74 (t 9.5)	68.8	CH	5.58	t (9.6)	16, 22	1, 15, 16, 23	14, 15, 21, 22, 30
18		16.8	1.26 (d 7.0)	1.23 (d 6.9)	16.5	CH_3	1.14	d (6.9)	4		2w, 3, 4, 5, 5-OH
19		13.8	1.93 (d 1.5)	1.90 s	13.5	CH_3	1.84	s	6, 9?	6, 7, 8	5, 9
20		21.5	0.97 (d 6.0)	0.98 (d 6.9)	20.9	CH_3	0.88	d (6.2)	13		12a, 12b, 14, 15, 16w?
21		58.1	3.39 s	3.36 s	56.8	CH_3	3.25	s		15	6,14, 17
22		123.3	5.14 (dd 9.5, 1)	5.11 (d 9.5)	123.6	CH	5.11	d (8.94)	17, 30	24,30	17, 24, 25w, 30
23		143.3			140.6	C_q					
24	ab	38.1	2.75, 2.81	2.70 m, 2.80 m	36.7	CH_2	2.68	m2H	25		22, 30
25		130.1	5.50 m	5.50 b m	129.2	CH	5.38	m	24, 26		24
26		127.4	5.58 m	5.6 b m	126.7	CH	5.49	very broad	25, 27ab		27a
27	a	39.0	2.36m	2.36 m	37.6	CH_2	2.37	dd (14.4, 7.6)	26	25, 26, 28	26
	b		2.52 m	2.54 m			2.25	dd (15.1, 6.9)	26	28, 29	
28		76.1			73.3	C_q					
29		181.1			177.7	C_q					
30		17.9	1.8 s	1.77 s	17.2	CH_3	1.68	s	22	22, 23, 24	17, 24, 22w
31		26.4	1.35 s	1.35 bs	25.5	CH_3	1.22	s		27, 28, 29	

[a] coupling constants based on coupling for H-3 and H-5 and H_3-18, signal overlapping; [b] From proton to carbon listed

Figure S3. ^{1}H NMR spectrum of Leiodolide A used in the study (d_4-methanol, 600 MHz).

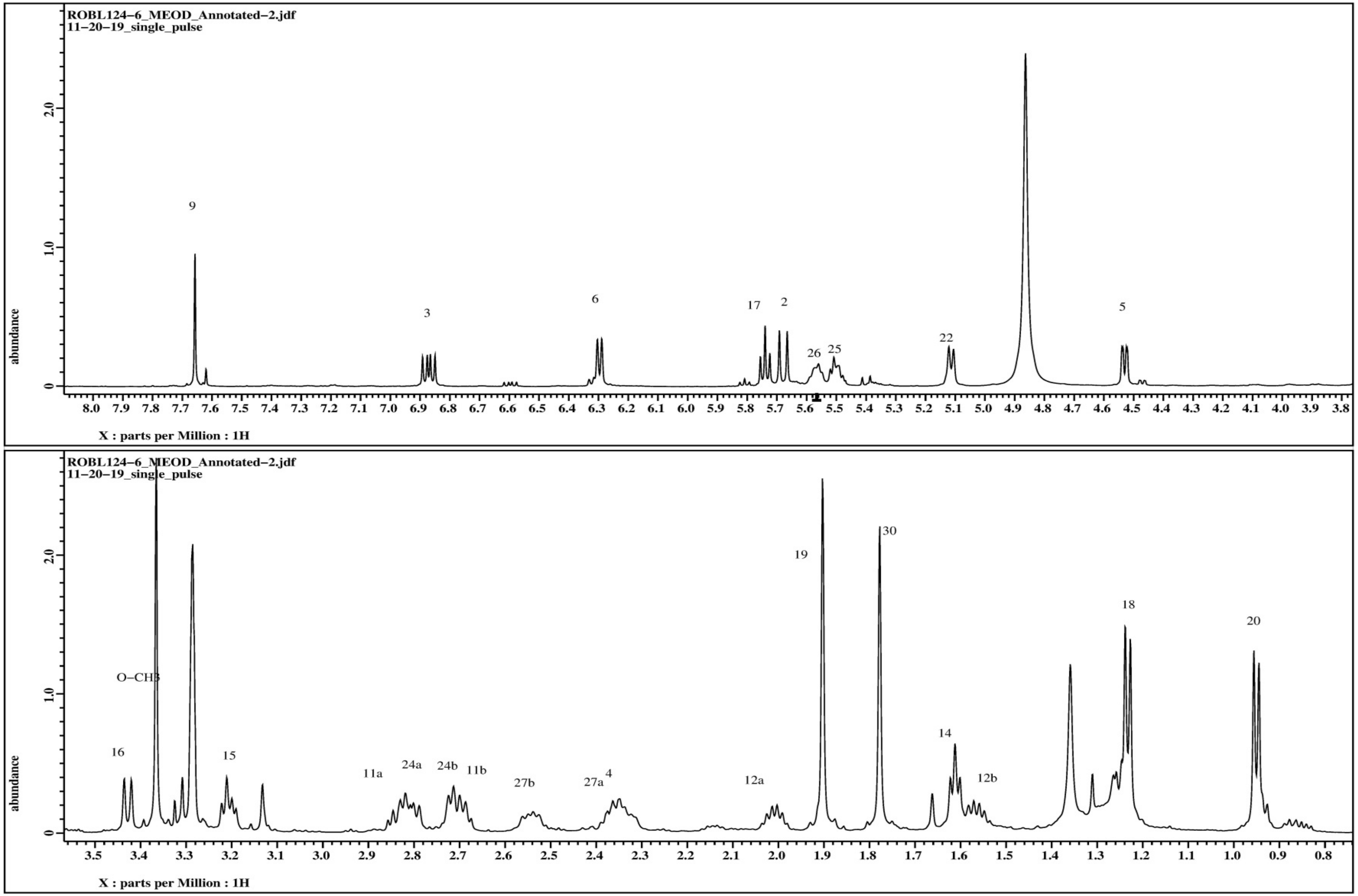

Figure S4. ^{1}H NMR spectrum of Leiodolide A used in the study (d_6-DMSO, 600 MHz).

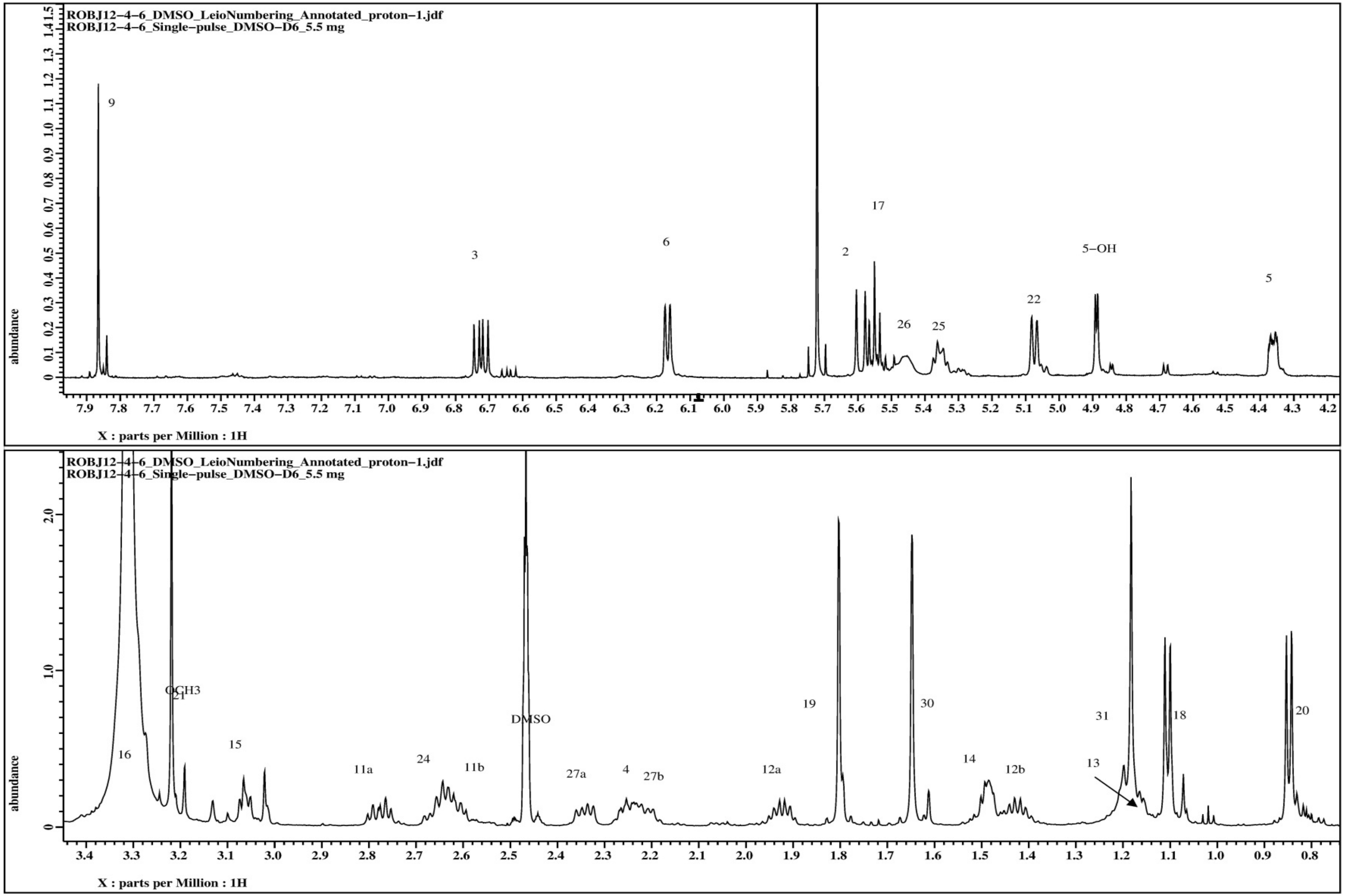

Figure S5. Comparison of ^{1}H NMR spectra of Leiodolide A before and after further purification by reverse phase HPLC. Top spectrum Leiodolide A as originally purified with all resonances. Bottom spectrum leidolide A after additional purification by semi-preparative C-18 HPLC, most side chain resonances are not visible (*d4*-methanol, 600 MHz).

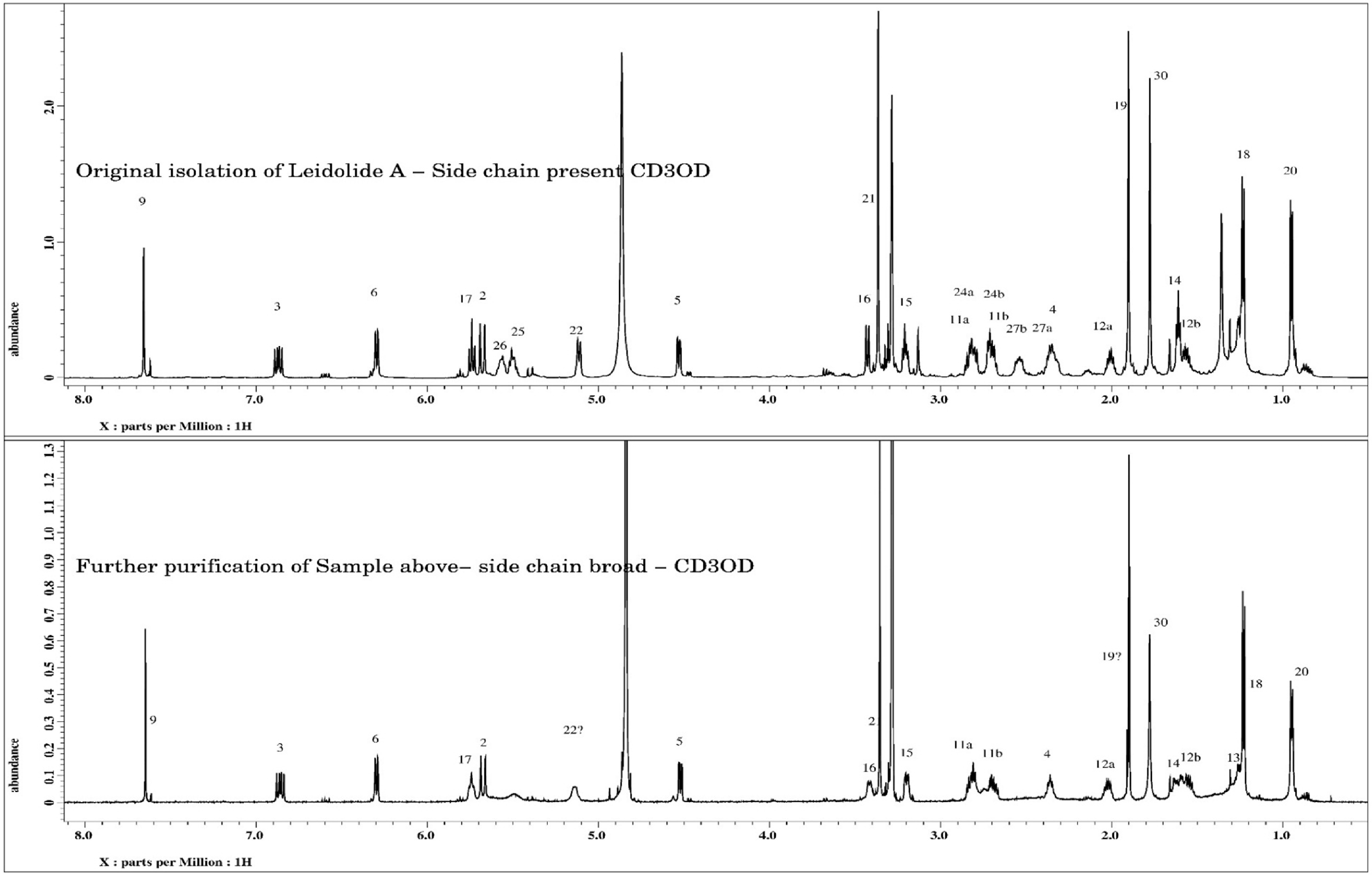

Figure S6. ^{13}C NMR spectrum of Leiodolide A used in the study (d_6-DMSO, 150 MHz).

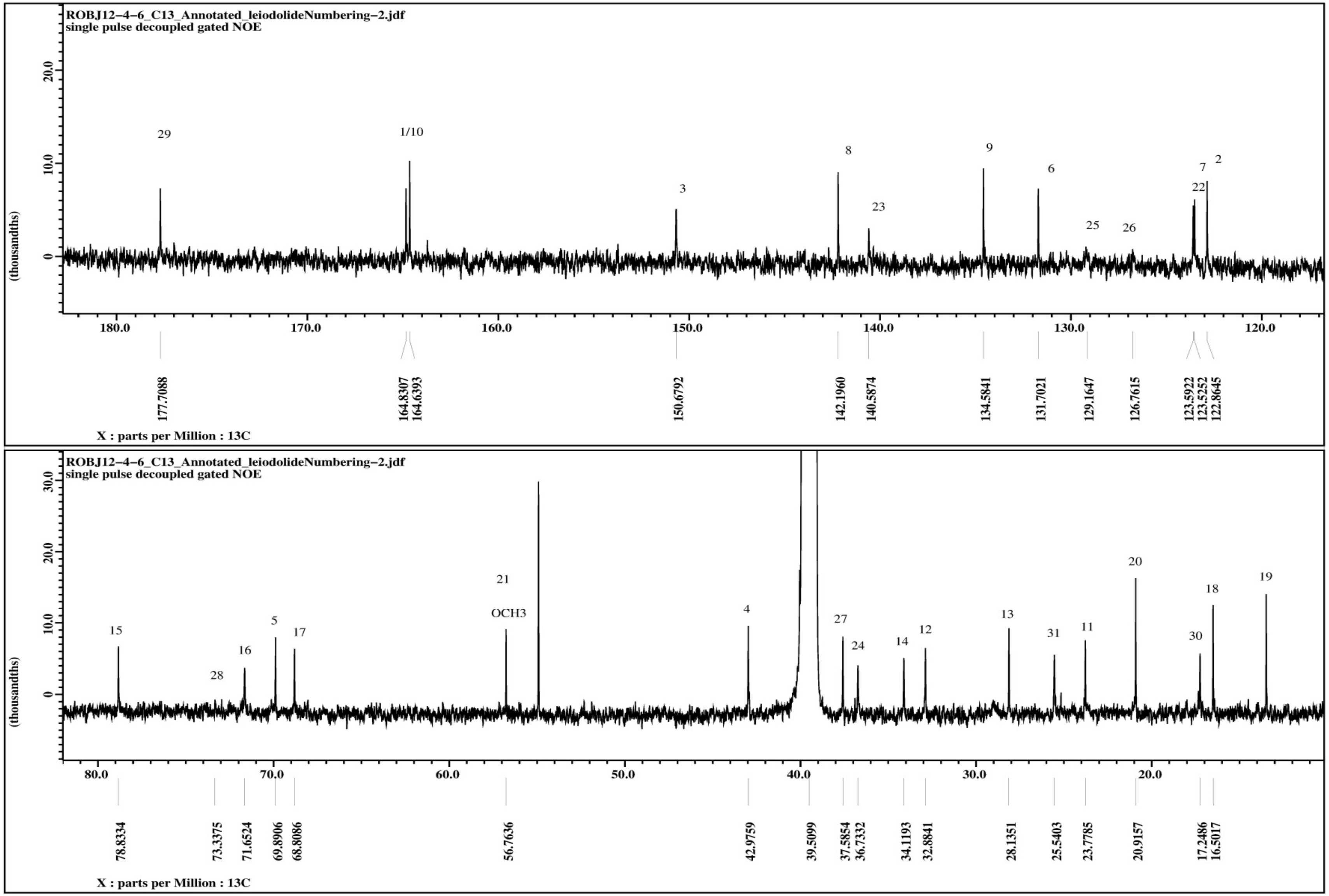

Figure S7. Edited g-HSQC spectrum of Leiodolide A used in the study (d_6-DMSO, 600 MHz).

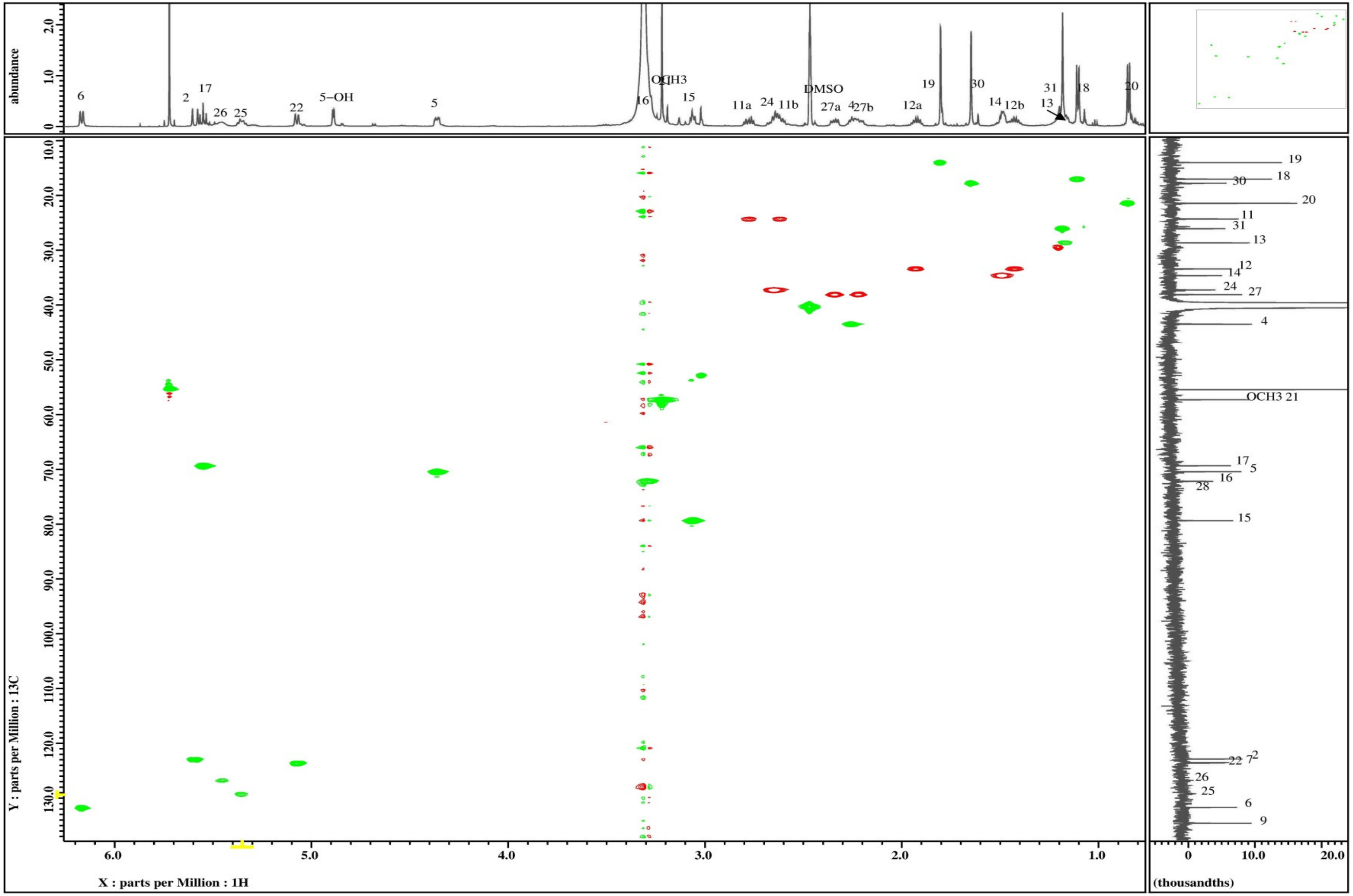

Figure S8. 2D-HMBC spectrum of Leiodolide A used in the study (d_6-DMSO, 600 MHz).

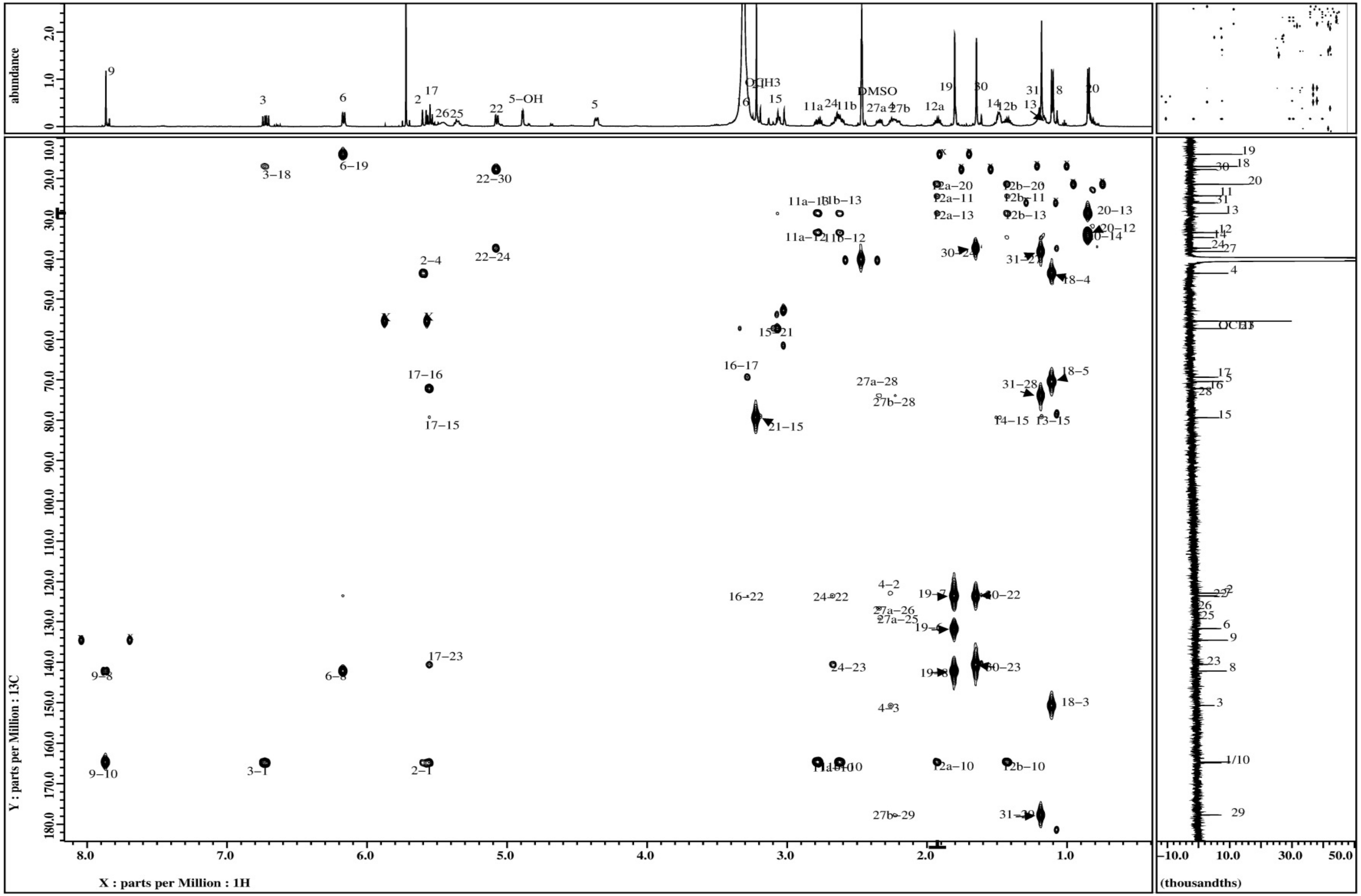

Figure S9. 2D-COSY spectrum of Leiodolide A used in the study (d_6-DMSO, 600 MHz).

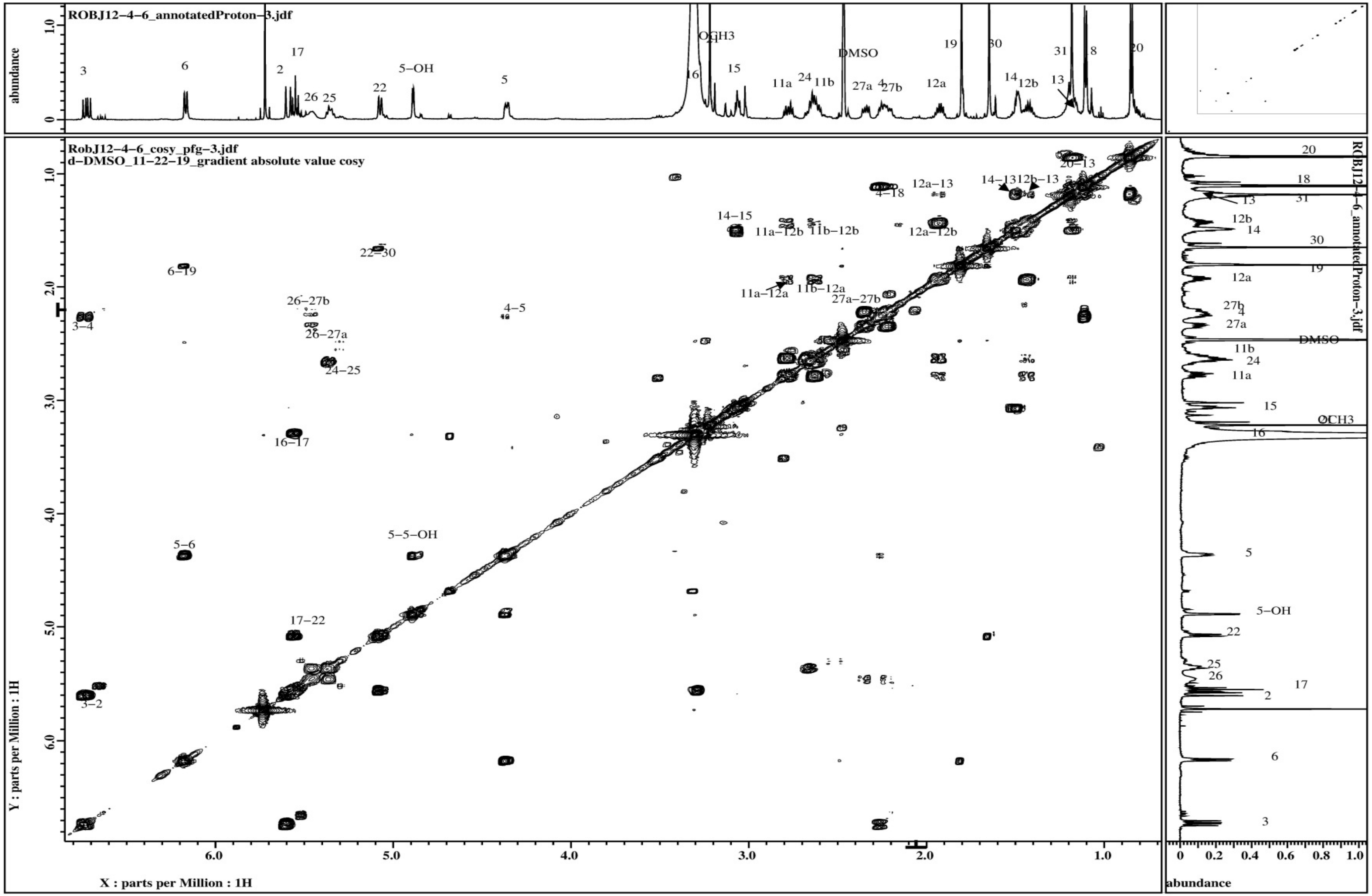

Figure S10. 2D-NOESY spectrum of Leiodolide A (**1**) used in the study (d_6-DMSO, 600 MHz).

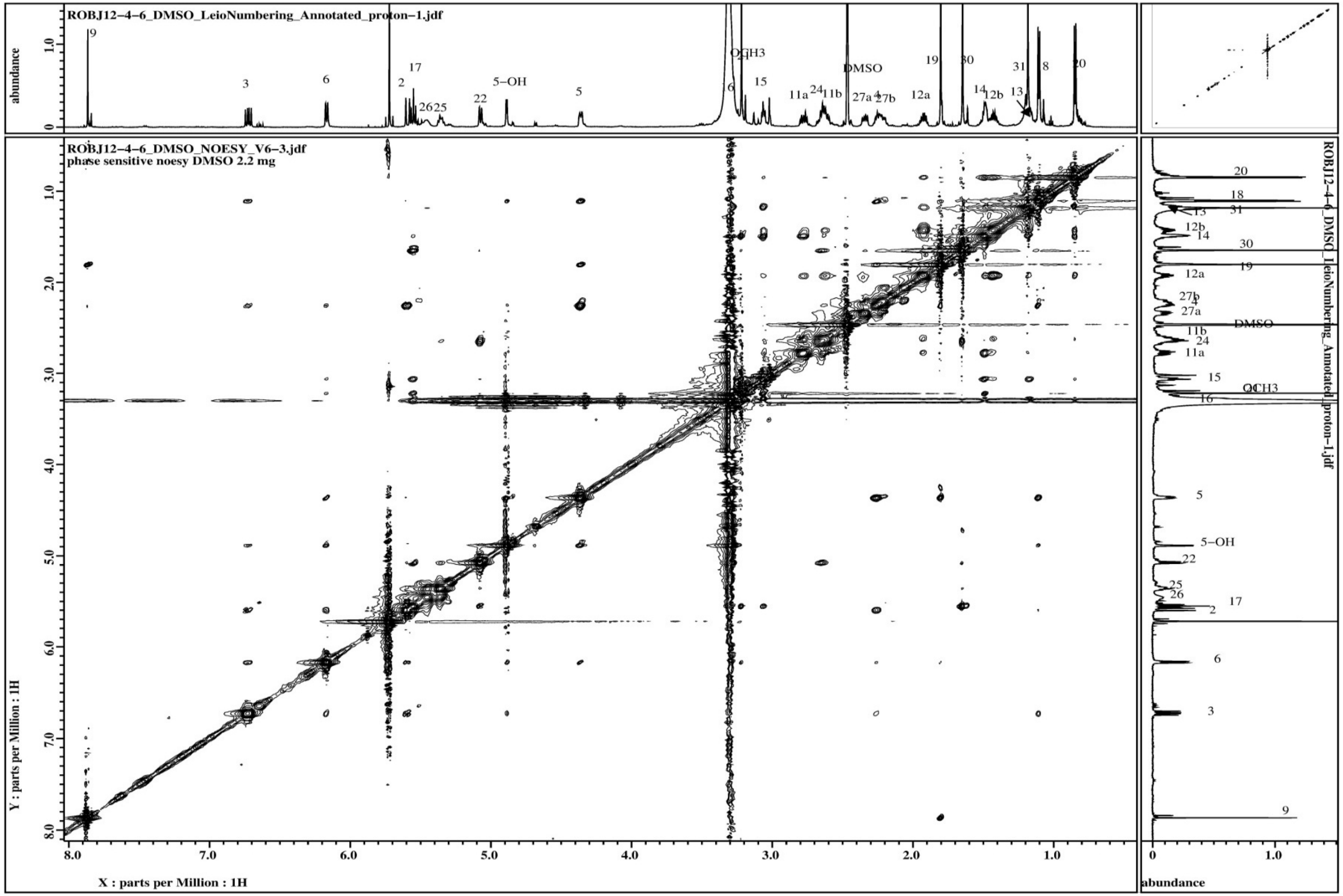

Figure S11. Expansion of 2D-NOESY spectrum of Leiodolide A used in the study (d_6-DMSO, 600 MHz).

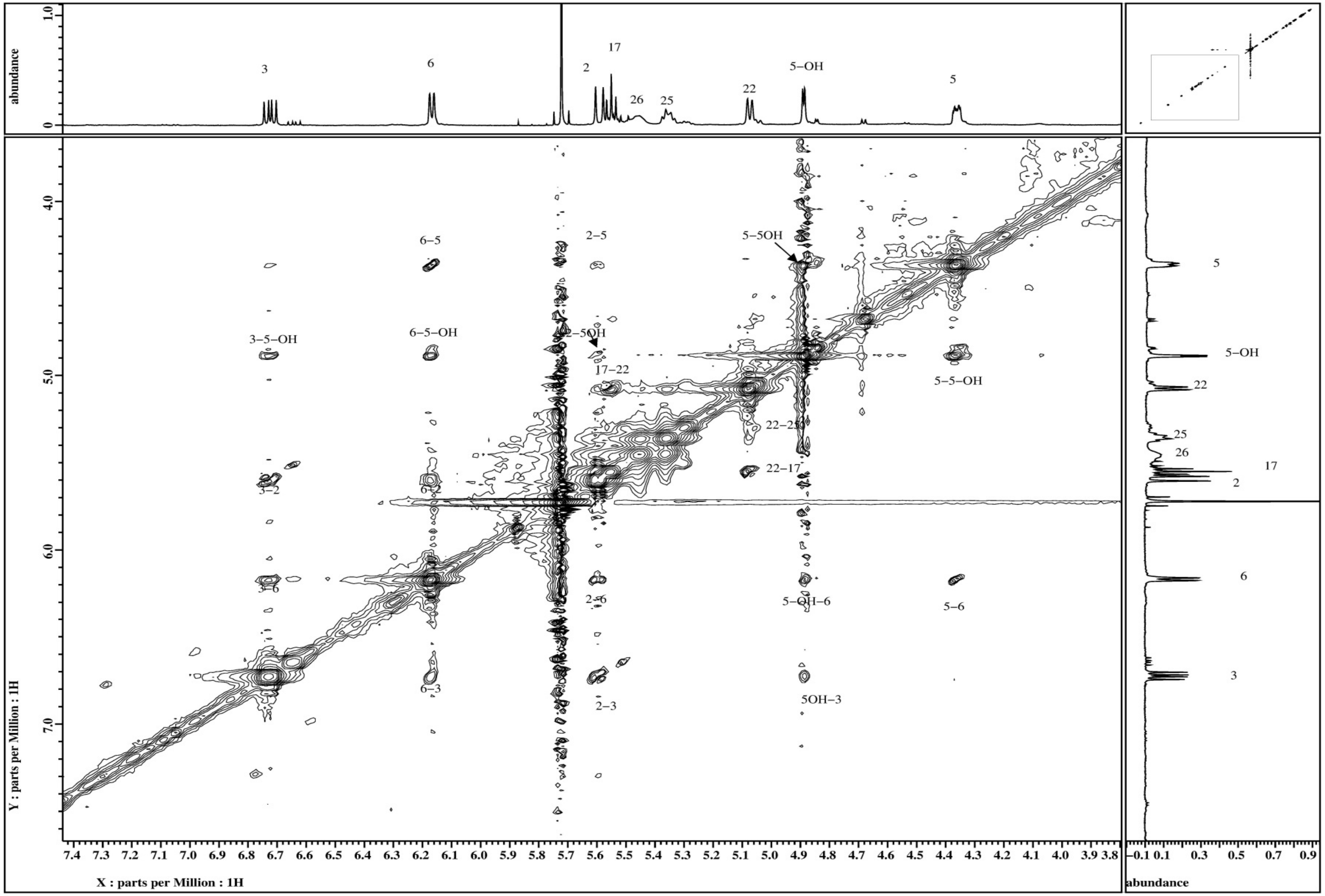

Figure S12. Expansion of 2D-NOESY spectrum of Leiodolide A used in the study (d_6-DMSO, 600 MHz).

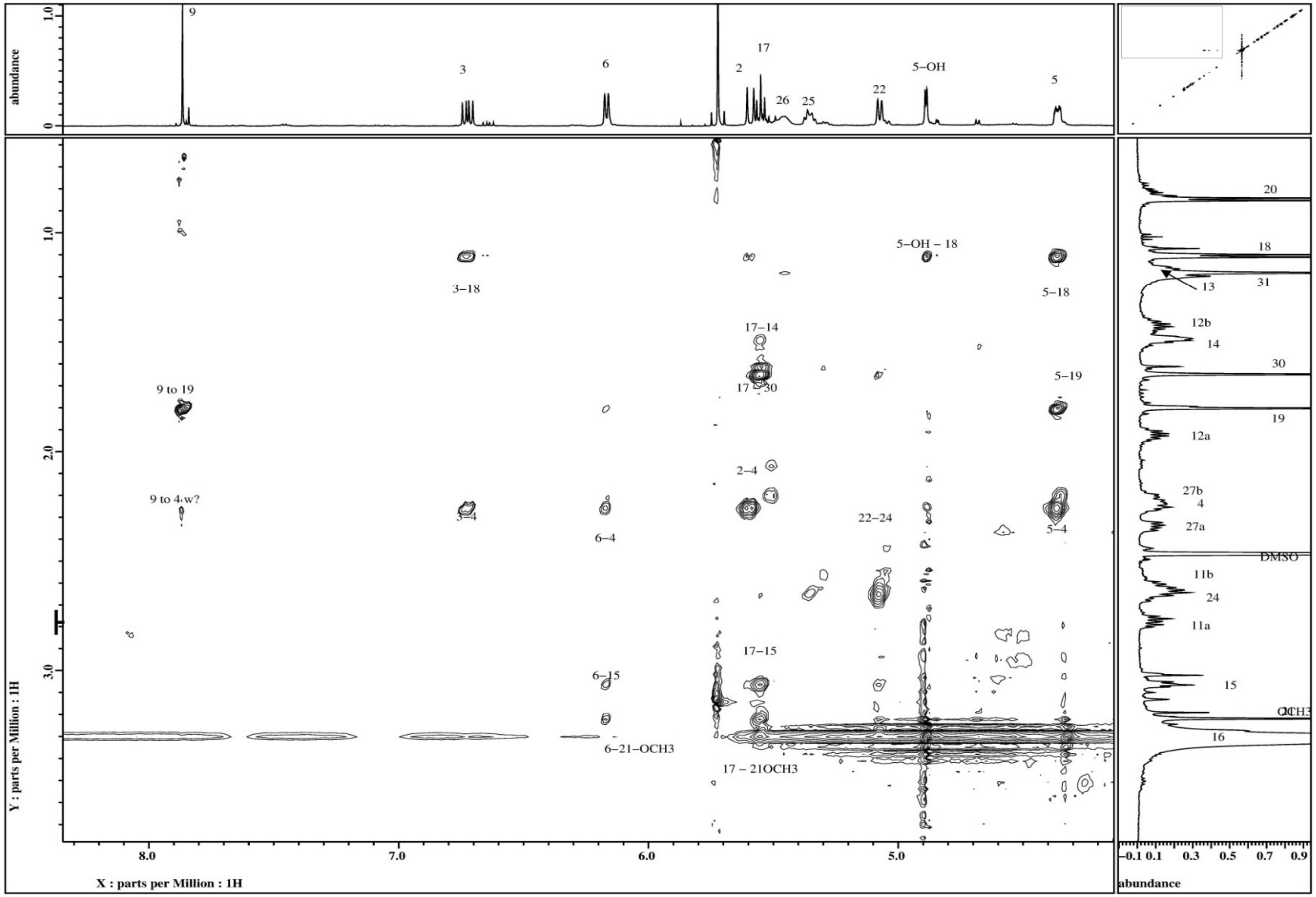

Figure S13. Expansion of 2D-NOESY spectrum of Leiodolide A used in the study (d_6-DMSO, 600 MHz).

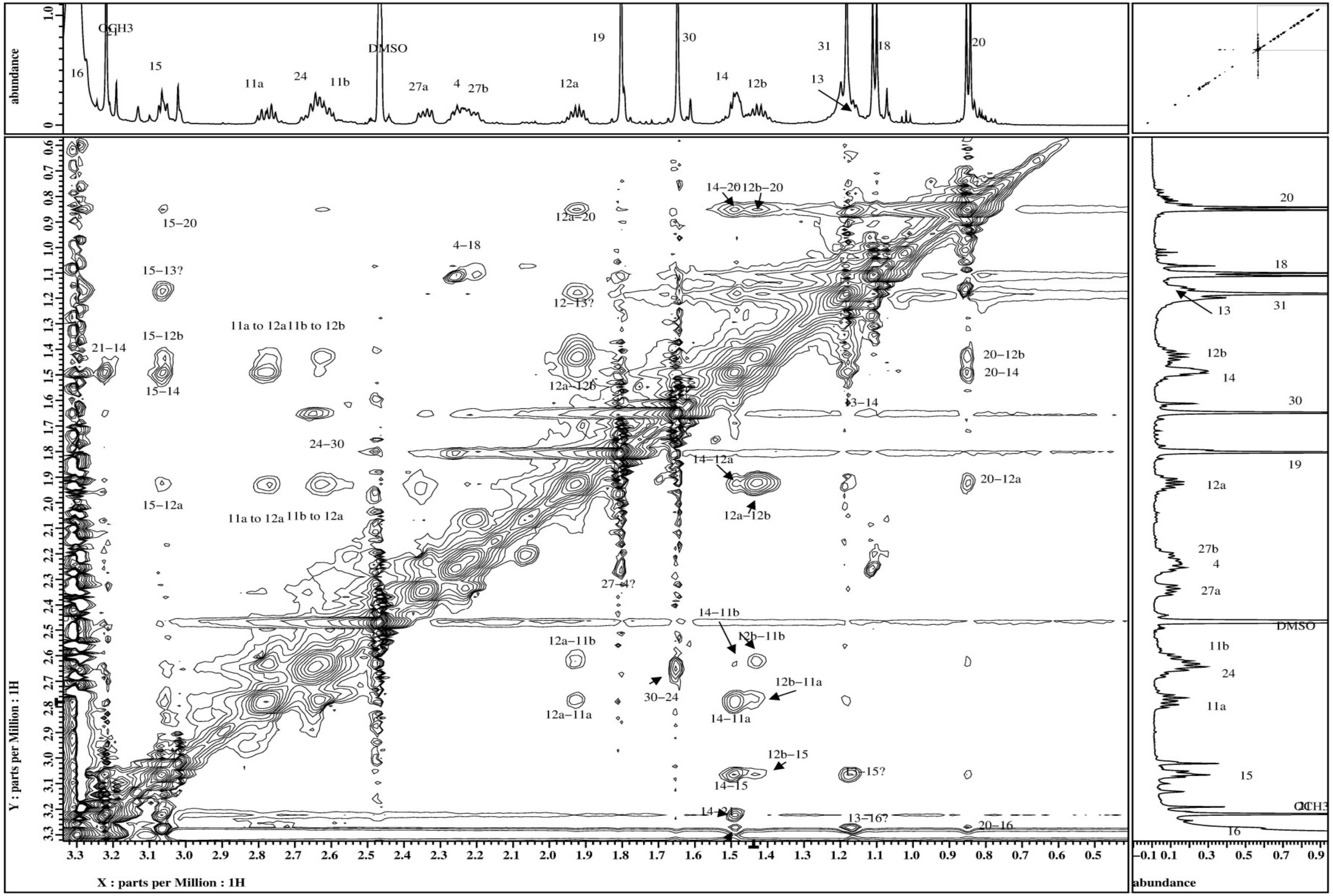

Figure S14. Expansion of 2D-NOESY spectrum of Leiodolide A used in the study (d_6-DMSO, 600 MHz).

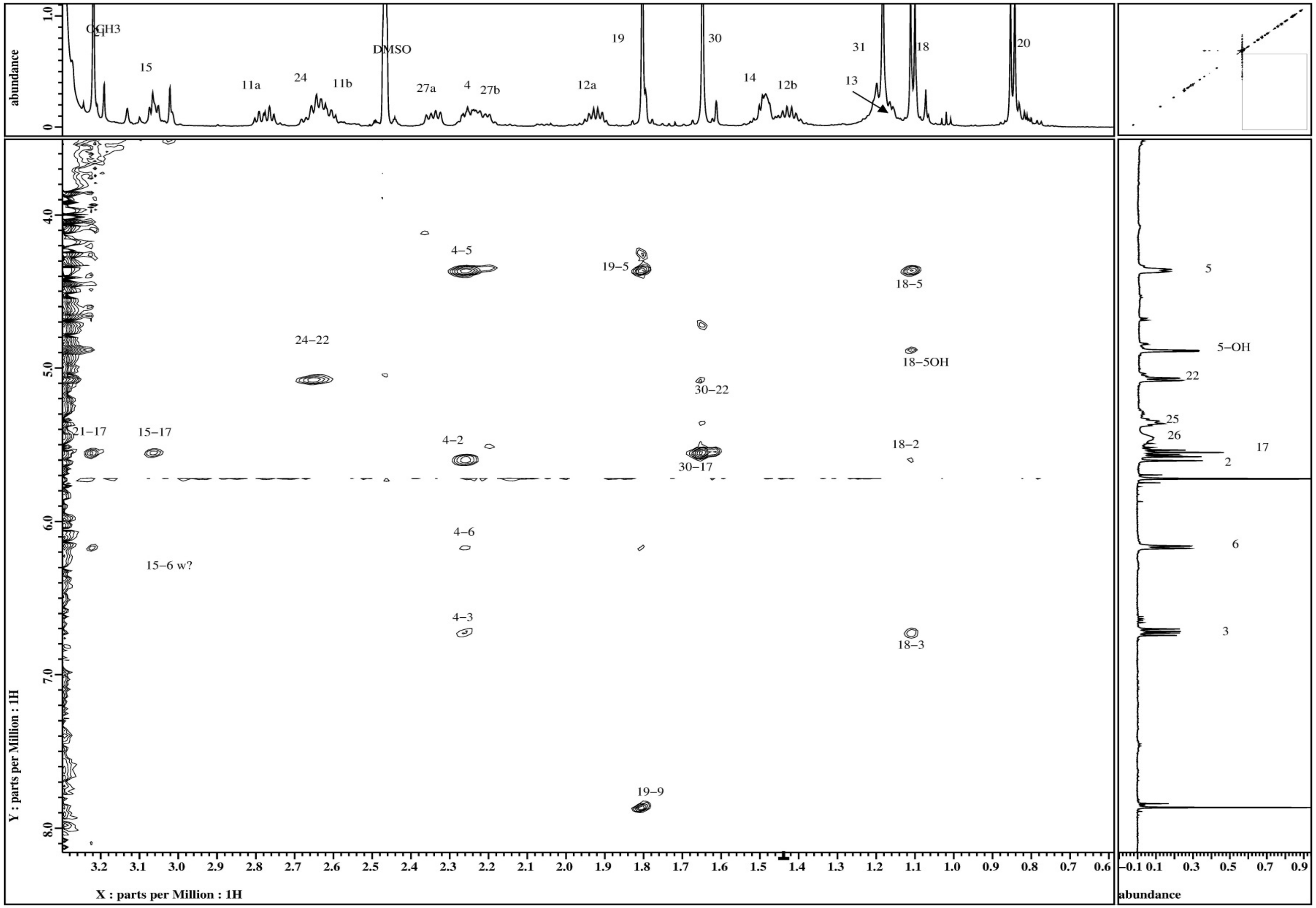

Figure S15. High resolution mass spectrum of Leiodolide A used in this study. Leiodolide A observed 598.2968 [M + H] + Calc'd 598.299204 $[M + Na]^+$ Δ = 0.0006

Elemental Compositions

D:\msAxel@LP Data\Priscilla data\RobJ12-4-6_PS+.txt

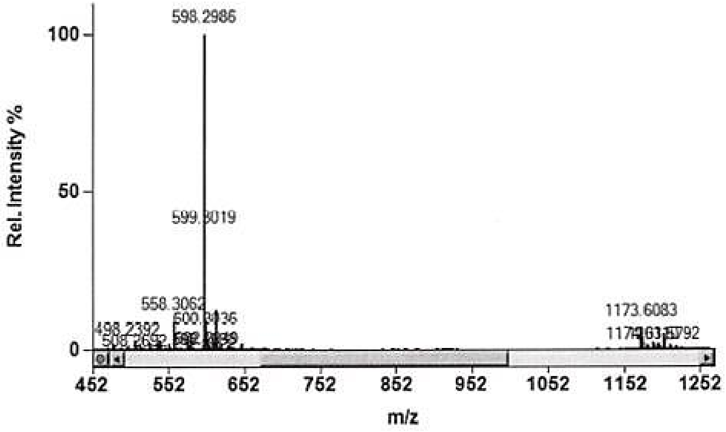

Mass Spectrum

Elemental Compositions
Element Limits: C 0/50 H 0/100 O 0/10 N 0/10 Na 1/1
Tolerance: 5 mmuEven or odd electron ion or both: Both
Electron correction: None.Charges: 1
Minimum unsaturation: -1Maximum unsaturation: 100

Calc. m/z	Abund %	mmu	DBE	Composition
598.293835	100.000	-4.69	6.0	C24H43O10N6Na1
598.295171	100.000	-3.35	11.0	C25H39O6N10Na1
598.296515	100.000	-2.01	10.5	C27H41O7N7Na1
598.297858	100.000	-0.66	10.0	C29H43O8N4Na1
598.299195	100.000	0.67	15.0	C30H39O4N8Na1
598.299202	100.000	0.68	9.5	C31H45O9N1Na1
598.300538	100.000	2.02	14.5	C32H41O5N5Na1
598.301874	100.000	3.35	19.5	C33H37O1N9Na1
598.301882	100.000	3.36	14.0	C34H43O6N2Na1
598.303218	100.000	4.69	19.0	C35H39O2N6Na1
598.294665	100.000	-3.86	23.5	C39H37N5Na1
598.296008	100.000	-2.51	23.0	C41H39O1N2Na1
